# Adjunctive Low-Dose Acetylsalicylic Acid in Bipolar Disorder: A Multicentre, Randomised, Double-Blind, Placebo-Controlled Phase II Trial (ASPIRIN BD)

**DOI:** 10.3390/jcm15218349

**Published:** 2026-10-27

**Authors:** Monika Dominiak, Anna Z. Antosik, Adam Gędek, Anna Krawczyk-Syguda, Sławomir Biedrzycki, Julita Kuczyńska, Michał Dermanowski, Paweł Mierzejewski

**Affiliations:** 1 Department of Pharmacology, Institute of Psychiatry and Neurology, 9 Sobieskiego Street, 02-957 Warsaw, Poland; 2 Department of Psychiatry, Collegium Medicum, Cardinal Stefan Wyszyński University, 01-938 Warsaw, Poland; 3 Department of Pharmacology and Third Department of Psychiatry, Institute of Psychiatry and Neurology, 02-957 Warsaw, Poland; 4 Holimedyk Mental Health Institute, 42-200 Częstochowa, Poland; 5 Regional Mental Health Hospital, 86-100 Świecie, Poland

**Keywords:** bipolar depression, bipolar disorder, acetylsalicylic acid, aspirin, anti-inflammatory, immunomodulation, randomised controlled trial, adjunctive treatment, relapse prevention

## Abstract

**Background/Objectives:** Bipolar depression remains difficult to treat. We evaluated the short-term antidepressant efficacy of adjunctive low-dose acetylsalicylic acid (ASA; 150 mg/day) over 2 months and its longer-term effect on relapse prevention over 12 months. **Methods:** ASPIRIN BD (EudraCT 2021-006635-25) was a multicentre, randomised, double-blind, placebo-controlled phase II trial at four Polish centres. Adults aged 18–60 years with a current bipolar depressive episode were randomised 1:1 to ASA or placebo for 12 months. The primary endpoint was the change in HAMD-17 score after 2 months, while the longer-term effect of ASA on relapse or clinical worsening was assessed over 12 months. **Results:** One hundred participants were randomised (50 per group). At month 2, ASA reduced HAMD-17 scores versus placebo (adjusted difference −4.24; 95% CI −7.20 to −1.28; *p* = 0.005; d = −0.51). Relapse or clinical worsening was less frequent with ASA (*p* = 0.026; NNT = 5). Depressive and bipolar remission rates were higher with ASA at month 6 (both *p* ≤ 0.003). Safety outcomes and adherence were comparable between groups. **Conclusions:** Adjunctive ASA 150 mg/day improved short-term depressive symptoms and reduced relapse or clinical worsening during a 12-month treatment period, with no clear safety signal. These findings warrant phase III confirmation.

## 1. Introduction

Bipolar disorder (BD) is a chronic, recurrent mood disorder associated with substantial functional impairment, reduced quality of life, elevated suicide risk, and premature mortality [1,2,3,4]. Depressive episodes account for the largest proportion of the symptomatic burden of BD and remain among its most difficult phases to treat effectively [2,5].

Importantly, most pharmacological evidence for depression comes from studies of major depressive disorder (MDD), which typically exclude patients with bipolar disorder [6,7]. As a consequence, treatment options for bipolar depression remain limited, and evidence supporting additional pharmacological strategies is inconsistent [8]. Regulatory indications for bipolar depression differ across jurisdictions and should be distinguished from evidence-based guideline recommendations. Regardless of the regulatory or guideline framework applied, suboptimal response to treatment, recurrence, and the long-term management of this chronic, relapsing disorder remain major clinical challenges worldwide. Given that patients with BD spend far more time in depressive than in manic states, this disparity between clinical need and available options represents a major, unresolved gap in psychiatric care and justifies the investigation of novel or repurposed therapeutic strategies [3,5,9].

Accumulating evidence suggests that immune dysregulation and neuroinflammatory processes may contribute to the pathophysiology of BD [10,11,12]. Reported abnormalities include alterations in peripheral inflammatory markers, including C-reactive protein (CRP), interleukin-6 (IL-6), and tumour necrosis factor-α (TNF-α), as well as mechanisms involving glial activation, blood–brain barrier dysfunction, oxidative stress, impaired neuroplasticity, and altered immune–brain signalling [11,12,13]. These findings are neither universal nor static: inflammatory abnormalities vary according to mood state, illness stage, medication exposure, metabolic factors, comorbid disorders, and other patient characteristics [12,14]. Consequently, BD may be better conceptualised as comprising partially distinct inflammatory and clinical phenotypes rather than a single, uniform inflammatory disorder [12,13]. This heterogeneity may be particularly relevant to bipolar depression. In a secondary analysis involving 266 individuals with bipolar depression, low-grade inflammation—defined as CRP concentrations above 3 mg/L—was reported in 70.9% of participants and was associated with anhedonia, fatigue, hypersomnia, and psychomotor retardation [15].

Interest has therefore grown in anti-inflammatory and immunomodulatory agents as adjunctive treatments for BD, including celecoxib, minocycline, N-acetylcysteine (NAC), and acetylsalicylic acid (ASA) [16,17,18]. However, results have been inconsistent. A network meta-analysis identified potentially beneficial effects for adjunctive NAC and selected anti-inflammatory strategies but also emphasised the limited certainty and heterogeneity of the available evidence [18]. Similarly, although some smaller studies suggested potential benefits of celecoxib augmentation in bipolar depression, the larger MINDCARE trial did not demonstrate superiority of celecoxib or minocycline over placebo [19,20]. These inconsistent findings reinforce the possibility that response to anti-inflammatory treatment is conditional on biological and clinical heterogeneity [14,15].

ASA is a particularly attractive candidate for repurposing because it is inexpensive, widely available, and acts through cyclooxygenase-dependent pathways relevant to inflammatory signalling. The 150 mg/day dose was selected as a pragmatic low-dose regimen on the basis of the available observational and clinical evidence. Pharmacoepidemiological studies associated chronic low-dose ASA exposure, particularly within the 75–150 mg/day range, with a lower risk of affective episodes or clinical deterioration, whereas higher-dose exposure did not show a comparable signal [9,21,22]. Previous interventional studies had also evaluated doses close to this range, including 162 mg/day, while a substantially higher dose of 1 g/day did not demonstrate superiority over placebo when used alone in bipolar depression [23,24]. These data did not establish an optimal psychiatric dose, but supported evaluation of a low-dose regimen rather than a conventional high-dose anti-inflammatory regimen. Nevertheless, ASA use requires attention to bleeding risk, gastrointestinal adverse effects, drug interactions, and individual contraindications, all of which warrant careful screening and monitoring in a psychiatric trial context. A previous systematic review identified preliminary evidence supporting a possible role for low-dose ASA in mood disorders while emphasising the scarcity of robust, adequately powered randomised trials [9]. Randomised evidence for ASA in bipolar depression remains inconsistent. A factorial trial of aspirin and/or minocycline yielded an exploratory signal [23], whereas a smaller factorial trial of aspirin and NAC provided mixed evidence [24]. Most recently, the A-Bipolar RCT found no benefit of adjunctive ASA 150 mg/day over placebo on subclinical mood instability, assessed using daily patient-reported ratings, in patients with newly diagnosed BD [25]. Thus, previous trials have differed in population, dose, and outcome assessment, and evidence combining clinician-rated outcomes during an acute bipolar depressive episode with long-term follow-up remains lacking.

Accordingly, the ASPIRIN BD trial was designed to evaluate adjunctive ASA 150 mg/day in patients experiencing a bipolar depressive episode. The trial assessed clinician-rated depressive symptom severity at 2 months as the short-term efficacy outcome, alongside longer-term clinical outcomes over 12 months, including relapse or clinical worsening, remission, global functioning and quality of life, and safety. This design was intended to address a persistent evidence gap regarding the efficacy of ASA both in the treatment of bipolar depression and in relapse prevention and long-term outcomes in this recurrent disorder.

## 2. Materials and Methods

### 2.1. Trial Design

ASPIRIN BD was a multicentre, randomised, double-blind, placebo-controlled, parallel-group, phase II clinical trial evaluating the efficacy and safety of adjunctive low-dose acetylsalicylic acid (ASA) added to standard pharmacotherapy in adult patients with bipolar depression. Participants were randomly assigned in a 1:1 ratio to receive ASA 150 mg/day or matching placebo for 12 months, in addition to their prescribed mood-stabilising treatment. The trial was conducted in four psychiatric centres in Poland, coordinated by the Institute of Psychiatry and Neurology (IPiN), Warsaw, with the participation of Nowowiejski Hospital (Psychiatric Hospital in Warsaw), the Regional Mental Health Hospital in Świecie, and Holimedyk Mental Health Institute in Częstochowa. The total per-patient duration was 13 months (up to 4 weeks of screening, 12 months of blinded intervention, and a 1-month blinded post-intervention safety visit). The trial was initially registered with the European Union Drug Regulating Authorities Clinical Trials Database—EudraCT (2021-006635-25, dated 3 December 2021) under Directive 2001/20/EC and was subsequently transitioned to the EU Clinical Trials Information System (CTIS) under Regulation (EU) No 536/2014, where it is registered as EU CT No 2024-516584-86-00 (authorised for Poland on 9 August 2024). The trial protocol was approved by the Bioethics Committee of the Institute of Psychiatry and Neurology, Warsaw (Resolution No 18/2022, dated 27 April 2022) and by the Office for Registration of Medicinal Products, Medical Devices and Biocidal Products (URPL; authorisation No UR/DBL/D/048/2023, dated 9 February 2023). It was conducted in accordance with the Declaration of Helsinki, ICH GCP E6(R2), and applicable Polish and European Union legislation.

### 2.2. Participants

Eligible participants were adults aged 18–60 years with a diagnosis of bipolar disorder (BD; ICD-10 code F31, both BD-I and BD-II) currently presenting with a depressive episode (ICD-10 F31.3–F31.5), receiving standard pharmacological treatment for BD. Recruitment was conducted in outpatient mental-health clinics and psychiatric wards. The diagnosis and current mood state were verified at the screening visit by a psychiatrist according to ICD-10 criteria. The presence of a current bipolar depressive episode (ICD-10 F31.3–F31.5) was required for eligibility; however, the duration of the current depressive episode was not collected as a separate structured variable. The baseline clinical characterisation focused on the longitudinal course of bipolar disorder, including illness duration, prior affective episodes, psychiatric hospitalisations, suicide-attempt history, and current pharmacotherapy.

Inclusion criteria were: (1) established diagnosis of BD (ICD-10 F31), verified by clinical interview; (2) current depressive episode (ICD-10 F31.3–F31.5); (3) age 18–60 years, of either sex; (4) ongoing treatment with at least one mood stabiliser recommended for BD, excluding valproates; (5) ability to provide written informed consent; (6) body mass index (BMI) between 18 and 40 kg/m^2^; and (7) for women of childbearing potential, a negative serum pregnancy test at screening and agreement to use highly effective contraception throughout the trial. Women taking carbamazepine as part of standard therapy were counselled on the use of non-hormonal contraception because of enzymatic induction.

Exclusion criteria were: (1) comorbid severe psychiatric disorders (schizophrenia, dementia, substance-use disorder, obsessive–compulsive disorder); (2) chronic use of ASA for somatic indications; (3) regular use of steroidal or non-steroidal anti-inflammatory drugs (occasional use permitted), methotrexate, digoxin, acetazolamide, antidiabetic agents (insulin or sulfonylureas), anticoagulants (coumarin derivatives, heparin, direct oral anticoagulants), thrombolytics, or antiplatelet agents (ticlopidine, clopidogrel); (4) valproate use within the 7 days preceding enrolment, given the risk of clinically relevant interactions with ASA; (5) known allergy or hypersensitivity to ASA or other NSAIDs; (6) pregnancy or breastfeeding; (7) clinically significant somatic comorbidities, including severe cardiovascular disease, clinically relevant ECG abnormalities (PR > 240 ms, QRS > 110 ms, QTc > 450 ms, ST depression > 2 mm, or ST elevation > 1 mm), severe obstructive pulmonary disease, untreated thyroid disease, HIV or hepatitis A/B/C, myocardial infarction within the previous 6 months, insulin-dependent diabetes, gout, active gastrointestinal disease or history of peptic ulceration or major gastrointestinal surgery, hepatic impairment (≥3× ULN in at least two of ALT/AST/LDH/alkaline phosphatase/bilirubin), or renal impairment (BUN ≥ 30 mg/dL, creatinine ≥ 2 mg/dL, or albuminuria); (8) receipt of any investigational medicinal product within 30 days prior to enrolment; (9) menorrhagia (menstrual blood loss > 80 mL per cycle or duration > 7 days); and (10) current use of non-pharmacological treatments (psychotherapy, electroconvulsive therapy, phototherapy, deep brain stimulation, or transcranial magnetic stimulation).

Recruitment began on 15 May 2023. The last screening visit occurred on 21 October 2025, and the last participant was randomised on 23 October 2025. The last scheduled treatment visit (month 12, V5) occurred on 30 April 2026, and the last post-treatment safety visit (month 13, V6) occurred on 30 May 2026. The database was closed on 2 September 2026. Four participants randomised relatively late in the recruitment period had not reached all scheduled 12- and 13-month assessments by the end of follow-up. All four had completed the month-2, month-4, and month-6 in-person assessments, with subsequent protocol-specified telephone follow-up where applicable. Participants whose later scheduled assessments had not yet become due were distinguished from those who discontinued participation or missed scheduled assessments.

### 2.3. Interventions

Participants received either enteric-coated ASA 150 mg or matching placebo orally, once daily, added to standard-of-care pharmacotherapy for BD, for 12 months. Placebo tablets were identical to ASA in size, appearance, taste, and smell. The 150 mg dose was chosen on the basis of epidemiological and observational evidence suggesting a beneficial effect of low-dose ASA on affective episodes at doses used for cardiovascular prevention.

Standard-of-care treatment was determined by the patient’s treating psychiatrist, independently of the investigator, and could include mood stabilisers (lithium, carbamazepine, lamotrigine) and second-generation antipsychotics (quetiapine, olanzapine, aripiprazole, risperidone), together with antidepressants during depressive episodes. Participants were required to be receiving at least one mood-stabilising treatment recommended for bipolar disorder, whereas concomitant antidepressant treatment was permitted but not required. No protocol-defined minimum period of stability of background pharmacotherapy before randomisation was specified. Background treatment was prescribed by the treating psychiatrist independently of the study, and changes were permitted in response to clinical deterioration and recorded during follow-up. Valproates were prohibited throughout the trial because of clinically relevant interactions with ASA. Investigators did not modify standard therapy; any changes made by the treating psychiatrist in response to clinical deterioration were recorded at each visit, together with all concomitant medications. Chronic use of steroidal or non-steroidal anti-inflammatory drugs (including additional aspirin), methotrexate, digoxin, acetazolamide, sulfonylureas, insulin, anticoagulants, thrombolytics, and non-aspirin antiplatelet agents was prohibited during the trial; short-term use of NSAIDs for acute conditions (infection, pain) was permitted. Adherence was assessed at months 2, 4, 6, and 12 by tablet count and the Medication Adherence Rating Scale (MARS), and was additionally monitored during the eight interim telephone visits by asking participants how many tablets they had taken and how many tablets remained.

### 2.4. Outcomes

The protocol prespecified separate primary outcomes for the short-term and long-term phases of the trial. The short-term primary outcome was change in HAMD-17 score from baseline to month 2, reflecting efficacy in the treatment of the current bipolar depressive episode. The long-term primary outcome was the occurrence of relapse or clinical worsening during months 4–12.

- Short-term primary outcome: change from baseline in the 17-item Hamilton Depression Rating Scale (HAMD-17) at 2 months.
- Long-term primary outcome: proportion of participants meeting predefined criteria for relapse or clinical worsening at months 4, 6, and 12. Relapse (in patients with prior sustained remission of ≥8 weeks) or worsening (in patients whose remission lasted < 8 weeks) was defined as any of the following: psychiatric hospitalisation for an affective episode; modification of pharmacological treatment because of clinical worsening (dose increase, addition of a mood stabiliser, or addition of an antidepressant); emergence of suicidal ideation or a suicide attempt, or a score > 2 on HAMD-17 item 3 (suicide); or a Clinical Global Impression—Improvement (CGI-I) score ≥ 5. Participants who had not achieved remission at month 2 (HAMD-17 > 7) were classified as non-remitters. Remission status was reassessed at subsequent visits. Sustained remission of at least 8 weeks was operationally defined as HAMD-17 ≤ 7 at both the month-2 and month-4 assessments. Participants who subsequently achieved remission were classified accordingly from that assessment onward. Relapse/clinical worsening was recorded when at least one of the prespecified composite-event criteria was met. The long-term composite outcome was assessed from month 4 onward; events occurring before the month-4 assessment did not contribute to this outcome.

Secondary outcomes were assessed at 2 months and again at 4, 6, and 12 months, and included: (i) rates of depressive-episode remission (HAMD-17 ≤ 7); (ii) rates of full BD remission (HAMD-17 ≤ 7 and Young Mania Rating Scale [YMRS] ≤ 7); (iii) rates of clinically significant improvement (≥50% reduction in HAMD-17 from baseline); (iv) CGI-I score change; (v) change in the Global Assessment of Functioning (GAF); and (vi) frequency of suicide attempts.

Safety outcomes included the incidence of adverse events (AEs) and serious adverse events (SAEs); clinically relevant changes in vital signs (heart rate ≥ 15 bpm, systolic BP ≥ 20 mm Hg, or diastolic BP ≥ 10 mm Hg from baseline); body-weight change ≥7% from baseline at month 12 and ≥7% between months 12 and 13 at the post-treatment assessment; ECG abnormalities (ST depression > 2 mm, ST elevation > 1 mm, or QTc > 450 ms during treatment); and mortality at 4, 6, 12, and 13 months. A protocol-defined panel of laboratory measurements was obtained at every centre-based visit, comprising haematological parameters (white blood cell count, red blood cell count, haemoglobin, and platelet count); bleeding time; coagulation parameters (D-dimer, international normalised ratio [INR], prothrombin time [PT], activated partial thromboplastin time [APTT], and thrombin time [TT]); lipid profile (total cholesterol, low-density lipoprotein [LDL], high-density lipoprotein [HDL], and triglycerides); hepatic parameters (alanine aminotransferase [ALT] and gamma-glutamyl transferase [GGTP]); renal parameters; and thyroid-stimulating hormone (TSH). Exploratory biomarkers, including C-reactive protein (CRP) and interleukin-6 (IL-6), will be analysed and reported separately in a subsequent publication.

### 2.5. Sample Size

A planned sample of 100 randomised participants (50 per arm) was based on the short-term primary outcome. The sample-size calculation was based on a clinically meaningful between-group difference of 3 HAMD-17 points, prespecified in the study protocol, and an approximate pooled standard deviation of 5 points, corresponding to a standardised effect size of Cohen’s d ≈ 0.60. With a two-sided α of 0.05 and 80% power, approximately 88 participants were required. To allow for a 12% dropout rate, the target was set at 100 randomised patients, with approximately 120 patients screened.

### 2.6. Randomisation and Blinding

Participants were randomised 1:1 to ASA or placebo using a computer-generated block-randomisation sequence with block sizes of 2, 4, or 6, stratified by centre and by baseline depression severity (HAMD-17 < 18 vs. ≥18). The randomisation sequence was generated by the external contract research organisation (CRO) responsible for development and management of the study eCRF, independently of the study investigators. Allocation was implemented centrally through an Interactive Web Response System (IWRS), which assigned each enrolled patient a unique identifier and dispensed the corresponding pre-labelled investigational medicinal product. Site pharmacies and staff received sealed, coded medication packs identical in appearance for ASA and placebo.

The trial was double-blind: both participants and investigators were blinded to treatment allocation. In addition, the statistician analysing the study results remained blinded to treatment allocation. Blinding was maintained by the visually and organoleptically identical presentation of ASA and placebo tablets and by centralised, code-based dispensing. Procedures for emergency unblinding in case of a safety concern were managed through the sponsor and the IWRS system.

### 2.7. Statistical Analysis

The statistical analysis plan was prespecified within the final study protocol (IPIN/ASPIRIN/BD, version 6.0, dated 28 February 2023), before recruitment and before any outcome analyses were performed. No changes were made to the protocol-specified statistical analyses after trial commencement, and all prespecified analyses were performed as planned. Two non-prespecified interim safety reviews were conducted under study blinding; no formal interim efficacy testing or efficacy-based stopping rule was used, and the reviews did not affect recruitment, sample size, study procedures, or the prespecified final analyses. Additional sensitivity and exploratory analyses introduced during peer-review revision are identified as such. The database was locked on 2 September 2026.

The short-term and long-term primary outcomes were analysed separately using the prespecified statistical methods, with two-sided statistical significance set at *p* < 0.05; no formal multiplicity adjustment or hierarchical testing procedure was prespecified.

### 2.8. Populations

Three analysis populations were defined:

- Intention-to-treat (ITT)—all randomised participants (N = 100). The ITT population was used for the long-term primary outcome and for the sensitivity analysis of the short-term primary endpoint.
- Modified intention-to-treat (mITT)—all randomised participants with an available month-2 (V2) HAMD-17 assessment; this population was used for the primary short-term efficacy analysis and short-term secondary efficacy analyses based on observed data.
- Safety population—all participants who received at least one dose of study medication (N = 100).

### 2.9. Handling of Missing Data

The primary short-term efficacy analysis used observed V2 data in the mITT population (n = 95). As a sensitivity analysis, the full ITT population (N = 100) was analysed using last observation carried forward (LOCF) for the five participants with missing V2 HAMD-17 assessments, in accordance with the protocol-specified missing-data approach. For the long-term primary outcome, the primary analysis was performed in the ITT population using non-responder imputation (NRI). Participants for whom the long-term composite outcome could not be determined because of missing follow-up data were conservatively classified as having experienced relapse or clinical worsening under the prespecified non-responder imputation assumption. Participants with an evaluable long-term composite status were classified according to the observed outcome. A complete-case analysis including only participants with evaluable long-term outcome data was performed as a sensitivity analysis. Denominators therefore differed by analysis: the NRI analysis included the full randomised population (50 participants per group), whereas the complete-case long-term analysis included 33 participants in the ASA group and 31 in the placebo group. Secondary visit-specific analyses used participants with available data at the respective assessment.

Secondary efficacy analyses used observed data without imputation. Long-term categorical outcomes and continuous secondary outcomes were analysed using available complete cases at each visit. V6 (month 13) was considered a post-treatment assessment and was not included in the primary long-term treatment analyses.

### 2.10. Baseline Assessments and Descriptive Analyses

At baseline (V1), demographic and clinical characteristics were recorded, including age, sex, education, employment status, place of residence, bipolar disorder subtype, age at illness onset, illness duration, previous psychiatric hospitalisations, history of suicide attempts, family history of bipolar disorder, concomitant psychotropic treatment, and somatic comorbidities. Baseline psychopathology and functioning were assessed using the HAMD-17, YMRS, CGI-Severity, and GAF. Physical and laboratory assessments included body weight, heart rate, complete blood count, inflammatory markers (CRP and IL-6), lipid profile, TSH, D-dimer, and INR. Baseline characteristics were summarised descriptively by treatment group. Continuous variables were presented as mean (SD) or median (IQR), as appropriate; baseline laboratory variables were presented as mean (SD), and categorical variables as n (%). For descriptive between-group comparisons, approximately normally distributed continuous variables were compared using Welch’s t-test, while non-normally distributed continuous variables were compared using the Mann–Whitney U test. Categorical variables were compared using Pearson’s χ^2^ test or Fisher’s exact test, as appropriate. Standardised mean differences (SMDs) were additionally reported as a measure of baseline balance. Baseline *p* values were considered descriptive only and were not used to assess the success of randomisation.

### 2.11. Short-Term Primary Analysis

The short-term primary endpoint was analysed using ANCOVA in the mITT population, with the V2 HAMD-17 score as the dependent variable, baseline HAMD-17 as a covariate, and treatment group as the fixed effect. Adjusted between-group differences with 95% confidence intervals and two-sided *p*-values were reported. Cohen’s d, calculated using the pooled standard deviation of change from baseline, was reported as a standardised effect size. A corresponding ANCOVA in the full ITT population using LOCF was performed as a sensitivity analysis. The magnitude of the standardised effect was interpreted using Cohen’s benchmarks (small ≈ 0.2, medium ≈ 0.5, large ≈ 0.8) and compared with published effect estimates for currently first-line pharmacotherapies for bipolar I depression. As an additional sensitivity analysis, the primary ANCOVA model was adjusted for the randomisation stratification factors (recruiting centre and baseline depression-severity stratum [HAMD-17 < 18 vs. ≥18]). Model assumptions were assessed by examination of residual distributions, variance homogeneity, and the treatment-by-baseline HAMD-17 interaction; because heteroscedasticity was detected, HC3 robust standard errors were additionally calculated.

### 2.12. Long-Term Primary Outcome

The long-term primary outcome was the occurrence of relapse or clinical worsening during the on-treatment period V3–V5 (months 4, 6, and 12). The primary analysis used the ITT population (N = 100) with non-responder imputation (NRI), whereby participants with missing long-term outcome data were classified as having experienced relapse or clinical worsening. Between-group proportions were compared using Pearson’s χ^2^ test without continuity correction, with Fisher’s exact test reported as a robustness check. A complete-case analysis including participants with evaluable long-term outcome data was performed as a sensitivity analysis. The absolute risk reduction (ARR) and the number needed to treat (NNT = 1/ARR) were reported as clinical effect-size measures.

### 2.13. Continuous Secondary Efficacy Outcomes

YMRS and GAF at V2 were analysed using ANCOVA, with the corresponding V2 score as the dependent variable, treatment group as the fixed effect, and the corresponding baseline score as a covariate. CGI at V2 was analysed without baseline adjustment, because the baseline CGI assessment used the Severity component, whereas post-baseline assessments used the Improvement component. Long-term continuous efficacy outcomes (HAMD-17, YMRS, and GAF) at V3, V4, and V5 were analysed separately using ANCOVA, with the score at the relevant visit as the dependent variable, treatment group as the fixed effect, and the corresponding V1 score as a covariate. CGI at V3, V4, and V5 was analysed without baseline adjustment. Analyses were performed on available complete cases without imputation. Adjusted marginal means, intervention effects, and two-sided *p*-values were reported for each visit.

As a supplementary longitudinal analysis of depressive symptom severity during treatment, post-baseline HAMD-17 scores at months 2, 4, 6, and 12 were analysed using a mixed model for repeated measures (MMRM). The model included treatment group, visit, and the treatment-by-visit interaction as fixed effects, with baseline HAMD-17 score included as a covariate. Visit was treated as a categorical variable, and within-participant correlation across repeated assessments was modelled using an appropriate covariance structure. All available post-baseline observations were included without imputation. This analysis was considered supplementary and exploratory.

### 2.14. Response, Remission, and Other Categorical Outcomes

At V2, the proportions of participants achieving clinical response (≥50% reduction in HAMD-17 from baseline), depressive remission (HAMD-17 ≤ 7), and bipolar remission (HAMD-17 ≤ 7 and YMRS ≤ 7) were analysed in observed mITT cases. For the long-term on-treatment visits V3–V5, these categorical secondary outcomes were analysed using available complete cases at each visit. Between-group comparisons used Pearson’s χ^2^ test without continuity correction, with Fisher’s exact test reported as a robustness check. V6 was considered a post-treatment assessment and was not included in the primary long-term treatment analyses. Exploratory analyses of CRP and IL-6 as potential biomarkers of treatment response will be reported separately in a subsequent publication.

### 2.15. Exploratory Tertiary Analysis: Somatic Comorbidity

An exploratory tertiary analysis examined whether baseline somatic comorbidity modified the clinical effect of ASA. Somatic comorbidity was coded as a binary variable (1 = at least one somatic comorbidity present, 0 = no somatic comorbidity); psychiatric comorbidities were not classified as somatic conditions. For continuous HAMD-17 outcomes, separate ANCOVA models at V2–V6 included treatment group, somatic comorbidity, the treatment-by-comorbidity interaction, and baseline HAMD-17 score as a covariate. For categorical clinical response and depressive remission, logistic regression models included treatment group, somatic comorbidity, the treatment-by-comorbidity interaction, and baseline HAMD-17 score. Analyses were based on observed data without imputation and were considered exploratory; no multiplicity adjustment was applied.

### 2.16. Safety Analyses

Safety analyses were performed separately for the 12-month treatment period and for the post-treatment period between months 12 and 13. Adverse events (AEs), serious adverse events (SAEs), and clinically relevant abnormalities in vital signs were analysed using all available safety data for the respective assessment period. Other safety outcomes were analysed among participants with at least one evaluable assessment of the relevant parameter during the analysed period. Safety outcomes included AEs, SAEs, clinically relevant abnormalities in vital signs, body-weight change ≥ 7%, ECG abnormalities, abnormalities in coagulation parameters, bleeding time, and protocol-defined laboratory abnormalities. Categorical safety outcomes were compared between treatment groups using Pearson’s χ^2^ test without continuity correction, with Fisher’s exact test used as a robustness check, particularly for sparse data. No imputation was applied to missing safety data.

### 2.17. Software, Error Control, and Reporting

Statistical analyses were performed using STATISTICA, version 17.0 (TIBCO Software Inc., Palo Alto, CA, USA). All tests were two-sided with α = 0.05. Effect estimates are reported with 95% confidence intervals where applicable. Reporting follows the CONSORT 2025 statement [26].

**Figure 1:**
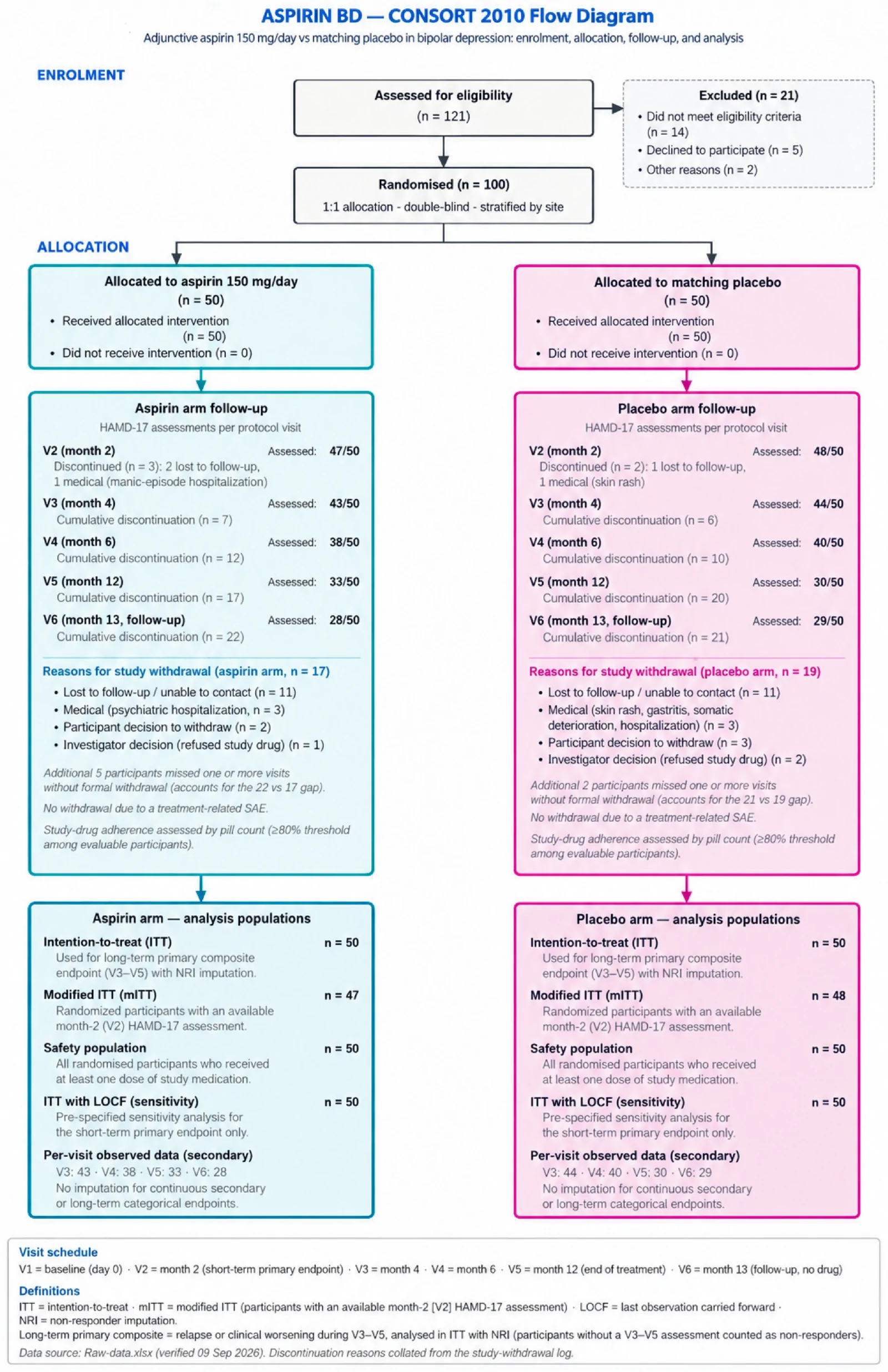
CONSORT 2025 flow diagram of participant enrolment, allocation, follow-up, and analysis in the ASPIRIN BD trial. Note: Four participants randomised late in the recruitment period completed the month-2 and month-6 in-person assessments but had not yet reached the scheduled month-12 and month-13 visits at study closure. These participants were not classified as discontinuations.

Gen AI was used to generate Figure 1 based on raw data. Figure 1 was subsequently reviewed and verified by the authors.

### 2.18. Study Visits and Assessments

The trial schedule comprised six on-site visits and eight telephone visits over 13 months, with a visit window of ±3 days. Screening (V1, week –4 to 0) included written informed consent, medical history, verification of eligibility, physical examination, vital signs, 12-lead ECG, serum pregnancy test (in women of childbearing potential), and a full laboratory panel including CRP and IL-6, together with the baseline HAMD-17, YMRS, CGI-S, GAF, and MARS assessments. Clinical global status was assessed using the Clinical Global Impression (CGI). At baseline (V1), the Severity component (CGI-S) was used to rate current illness severity, whereas at subsequent visits the Improvement component (CGI-I) was used to assess clinical change relative to baseline. Global functioning was assessed using the Global Assessment of Functioning (GAF), recorded in 10-point ranges across the 0–100 scale. For statistical analysis, these ranges were converted to ordered numerical categories according to the prespecified coding scheme used in the dataset (e.g., 11–20 = 1, 21–30 = 2, 31–40 = 3, 41–50 = 4, 51–60 = 5, 61–70 = 6, 71–80 = 7, 81–90 = 8, and 91–100 = 9), with higher coded values indicating better global functioning. Randomisation and first dispensing of investigational medication took place at baseline. On-site visits at months 2, 4, 6, and 12 (visits V2–V5, respectively) repeated the clinical rating scales, safety assessments, laboratory tests, ECG, and adherence measures. Telephone visits at months 1, 3, 5, 7, 8, 9, 10, and 11 included assessment of changes in the participant’s mental state, as well as collection of adverse-event data, home pregnancy-test results, and adherence information. The month-2 visit (V2) corresponds to the short-term primary endpoint. Study medication was discontinued at month 12, and a final on-site safety visit was performed one month later (month 13, V6) to assess post-discontinuation safety. Specialist consultations were offered to any participant reporting adverse events or laboratory or ECG abnormalities possibly related to trial participation, including for up to 3 months after unblinding (after V6) for participants who received ASA.

### 2.19. Patient and Public Involvement

Patients and members of the public were not involved as research partners in the design, conduct, analysis, or reporting of the study. Recruitment information was disseminated through patient-facing websites and relevant patient organisations.

### 2.20. Ethics, Funding, and Oversight

The trial protocol, informed-consent documents, and related materials were approved by the Bioethics Committee of the Institute of Psychiatry and Neurology, Warsaw (Resolution No 18/2022, dated 27 April 2022) and by the URPL (authorisation No UR/DBL/D/048/2023, dated 9 February 2023) prior to enrolment. Following the transitional provisions of Regulation (EU) No 536/2014, the trial was subsequently transitioned from the EudraCT (2021-006635-25) framework to CTIS and re-authorised for Poland on 9 August 2024 under EU CT No 2024-516584-86-00. Written informed consent was obtained from every participant before any protocol-mandated procedure. The trial was performed by the Institute of Psychiatry and Neurology (Warsaw) and funded by the Polish Medical Research Agency, grant no. 2021/ABM/02/00008-00. Sponsor and investigator liability insurance was in place in accordance with Polish regulations. Adverse events were recorded and reported according to ICH GCP and Polish pharmaceutical law; serious adverse events were reported to the sponsor within 24 h (or the next working day) for life-threatening or unexpected events, and suspected unexpected serious adverse reactions (SUSARs) were reported to URPL and the Bioethics Committee within 15 days (7 days for fatal or life-threatening events). Annual Development Safety Update Reports (DSURs) were submitted to URPL and the Bioethics Committee. Monitoring was performed by Masha Regulatory Service (contract research organisation) on behalf of the sponsor. The study was also subject to an audit by the funding body, the Medical Research Agency, conducted between September 2025 and February 2026. The clinical conduct of the trial, including compliance with applicable regulatory requirements and study procedures, was assessed as appropriate (Inspection Protocol no 2021/ABM/02/00008/K/01). Trial data are the property of the sponsor and will be reported in accordance with the CONSORT 2025 statement [26].

## 3. Results

### 3.1. Participant Flow

Between 15 May 2023 and 23 October 2025, 121 participants were screened for eligibility and 100 were enrolled and randomised (ASA 50, placebo 50). Study follow-up continued until 31 May 2026. By V2, five participants (5%) had discontinued the study: three in the ASA arm (two lost to follow-up and one for a medical reason [hospitalisation due to a manic episode]) and two in the placebo arm (one lost to follow-up and one for a medical reason [skin rash]). At study closure, four participants had not yet reached the scheduled month-12 and month-13 assessments because of late randomisation; all four had completed the month-2, month-4, and month-6 in-person assessments. Participant flow is depicted in Figure 1.

### 3.2. Baseline Characteristics

Baseline demographic and clinical characteristics of the two treatment arms were comparable (Table 1). The mean age was 39.2 ± 11.5 years in the ASA arm and 38.8 ± 10.7 years in the placebo arm; 64.0% and 60.0% were women, respectively. Mean (SD) HAMD-17 at baseline was 15.92 (6.91) in the ASA arm and 16.70 (5.88) in the placebo arm. Bipolar I disorder was present in 74.0% versus 60.0% of participants in the ASA and placebo groups, respectively (*p* = 0.202). Baseline psychotropic treatment was also comparable, including lithium (24.0% vs. 22.0%), lamotrigine (64.0% vs. 72.0%), any second-generation antipsychotic (72.0% vs. 60.0%), and any antidepressant (50.0% vs. 56.0%). Any somatic comorbidity was present in 42.0% of the ASA arm and 36.0% of the placebo arm (*p* = 0.682). Baseline laboratory parameters did not differ significantly between groups (all *p* > 0.05). Detailed baseline characteristics are provided in Table 1. Additional information on baseline depressive symptom severity, the longitudinal clinical history of bipolar disorder, baseline concomitant pharmacotherapy, and clinically relevant treatment changes during follow-up is provided in Supplementary Table S1.

### 3.3. Short-Term Efficacy Analysis at Month 2

#### 3.3.1. Primary Outcome—Change in HAMD-17 Score at Month 2

##### Primary Analysis

Of the 100 randomised participants, five (5.0%) had no HAMD-17 assessment at month 2 (three in the intervention group and two in the control group) and were therefore not included in the primary analysis. The primary short-term efficacy analysis was performed using ANCOVA in the mITT population (n = 95), with the month-2 HAMD-17 score as the dependent variable and baseline HAMD-17 score as a covariate. A significant intervention effect was observed, F(1,92) = 8.11, *p* = 0.005, while baseline HAMD-17 score was also significantly associated with the month-2 outcome, F(1,92) = 19.41, *p* < 0.001.

Mean HAMD-17 change was −7.85 (SD 6.21) with aspirin versus −3.88 (SD 9.04) with placebo; adjusted between-group difference −4.24 [95% CI −7.20 to −1.28], *p* = 0.005, Cohen’s d = −0.51. Although the point estimate exceeded the prespecified 3-point threshold for clinical relevance, the confidence interval included effects smaller than this threshold, indicating some uncertainty regarding the magnitude of the clinically relevant benefit. Detailed efficacy results are presented in Table 2.

Adjustment for the randomisation stratification factors produced a virtually unchanged treatment estimate (adjusted difference −4.27 HAMD-17 points, 95% CI −7.33 to −1.20; *p* = 0.007). The treatment-by-baseline HAMD-17 interaction was not significant (*p* = 0.252), supporting the homogeneity-of-regression-slopes assumption. Although heteroscedasticity was detected, the treatment effect remained significant using HC3 robust standard errors (95% CI −7.60 to −0.93; *p* = 0.013).

##### Sensitivity Analysis

Sensitivity analysis on the intention-to-treat population (N = 100) confirmed the primary result. Analysis of covariance (ANCOVA) with baseline HAMD-17 as a covariate and last-observation-carried-forward (LOCF) imputation of the five missing V2 assessments was used. A significant intervention effect was observed, F(1,97) = 7.31, *p* = 0.008, while baseline HAMD-17 score was also significantly associated with the month-2 outcome, F(1,97) = 27.78, *p* < 0.001.

Mean HAMD-17 change from baseline was −7.38 (SD 6.31) in the aspirin group and −3.72 (SD 8.88) in the placebo group, corresponding to an adjusted between-group difference of −3.97 HAMD-17 points [95% CI −6.88 to −1.06], *p* = 0.008, Cohen’s d = −0.48. The treatment estimate was similar in the LOCF sensitivity analysis; however, this does not exclude bias arising from missing data.

#### 3.3.2. Secondary Outcomes

##### Response and Remission Rates—Depressive Episode

The proportion of patients achieving response (≥50% reduction in HAMD-17 from baseline) and remission (HAMD-17 ≤ 7) was compared between treatment groups using Pearson χ^2^ tests without continuity correction, with Fisher’s exact tests reported as robustness checks.

At V2, response rates were higher in the ASA group than in the placebo group (60% vs. 42%), although the difference did not reach statistical significance (χ^2^ = 3.05, *p* = 0.081; Fisher’s exact *p* = 0.102). Remission rates were also higher in the ASA group than in the placebo group (57% vs. 38%), although this difference did not reach statistical significance (χ^2^ = 3.80, *p* = 0.052; Fisher’s exact *p* = 0.065).

##### Bipolar Remission

Bipolar remission, defined as HAMD-17 ≤ 7 and YMRS ≤ 7 at V2, was achieved by 57.4% of participants in the ASA group and 37.5% in the placebo group (27/47 vs. 18/48). The between-group difference did not reach statistical significance (χ^2^ = 3.79, *p* = 0.052; Fisher’s exact *p* = 0.065).

##### Change in Young Mania Rating Scale Score at Month 2

For YMRS, ANCOVA showed no significant effect of intervention on the month-2 score, F(1,92) = 0.09, *p* = 0.770. Baseline YMRS score was also not significantly associated with the month-2 outcome, F(1,92) = 0.27, *p* = 0.605. The adjusted mean YMRS scores at V2 were 1.32 in the ASA group and 1.12 in the placebo group.

Change in Clinical Global Impression and Global Assessment of Functioning scores at month 2

Secondary continuous outcomes (YMRS, CGI, GAF) were analysed by ANCOVA on the mITT population.

For CGI, ANCOVA showed no significant effect of intervention on the month-2 score, F(1,92) = 0.45, *p* = 0.504. Baseline CGI score was also not significantly associated with the month-2 CGI outcome, F(1,92) = 0.90, *p* = 0.344.

For GAF, the intervention effect was not statistically significant, F(1,92) = 1.70, *p* = 0.195. In contrast, baseline GAF score was strongly associated with the month-2 GAF outcome, F(1,92) = 15.07, *p* < 0.001.

##### Suicide Attempts at V2

No suicide attempts were reported in either the ASA or placebo group at V2.

### 3.4. Long-Term Efficacy Analysis at Months 4, 6, and 12

#### 3.4.1. Primary Outcome

Among participants with evaluable long-term composite outcome data, relapse or clinical worsening was observed in 14 of 33 participants (42.4%) in the ASA group and 22 of 31 (71.0%) in the placebo group. The long-term composite outcome could not be determined in 17 participants in the ASA group and 19 participants in the placebo group because of missing follow-up data. In the prespecified full ITT analysis using non-responder imputation (NRI), these participants were conservatively classified as having experienced relapse or clinical worsening, yielding event rates of 31 of 50 (62.0%) in the ASA group and 41 of 50 (82.0%) in the placebo group. The between-group difference was statistically significant (Pearson χ^2^ = 4.96, *p* = 0.026; two-sided Fisher’s exact *p* = 0.044). The absolute risk reduction was 20.0 percentage points (95% CI 2.8 to 37.2), corresponding to a number needed to treat (NNT) of 5; the odds ratio was 0.36 (95% CI 0.14 to 0.90). This NNT refers to the prespecified ITT analysis incorporating non-responder imputation and should therefore not be interpreted as an estimate based solely on observed relapse or clinical-worsening events. The complete-case sensitivity analysis, restricted to participants with evaluable long-term composite outcome data, also showed a between-group difference (Pearson χ^2^ = 5.29, *p* = 0.021; two-sided Fisher’s exact *p* = 0.026; ARR = 28.6 percentage points; NNT ≈ 4). Results for the composite endpoint are summarised in Table 2. Descriptively, the most frequent components of the long-term composite endpoint were psychiatric medication intensification and clinically significant worsening on the CGI. Psychiatric medication intensification occurred in 9/50 (18.0%) participants in the ASA group and 14/50 (28.0%) in the placebo group, while CGI-I ≥ 5 was observed in 11/50 (22.0%) and 22/50 (44.0%), respectively. Psychiatric hospitalisation for an affective episode occurred in one participant in the ASA group and none in the placebo group. No suicide attempts were recorded in either group during the long-term assessment period.

#### 3.4.2. Secondary Outcomes

##### Long-Term Efficacy—HAMD-17

At V3 (month 4), ANCOVA showed no statistically significant intervention effect on HAMD-17, F(1,84) = 3.43, *p* = 0.067; baseline HAMD-17 was significantly associated with the V3 score, F(1,84) = 7.27, *p* = 0.008. Adjusted mean HAMD-17 scores were 6.28 in the ASA group and 8.80 in the placebo group. At V4 (month 6), the intervention effect was significant, F(1,75) = 12.08, *p* < 0.001, with adjusted means of 4.69 versus 9.69, respectively; baseline HAMD-17 was not significant, *p* = 0.085. At V5 (month 12), the intervention effect did not reach statistical significance, F(1,60) = 3.25, *p* = 0.077, with adjusted means of 4.73 in the ASA group and 7.60 in the placebo group. The largest numerical between-group separation in the visit-specific analyses was observed at month 6.

In the supplementary longitudinal analysis of HAMD-17 scores across months 2, 4, 6, and 12, the treatment-by-visit interaction was not statistically significant (*p* = 0.385), providing no evidence that the magnitude of the treatment effect differed significantly across visits. Model-estimated between-group differences (ASA minus placebo) were −4.37 points (95% CI −7.01 to −1.73) at month 2, −2.52 (95% CI −5.26 to 0.23) at month 4, −4.96 (95% CI −7.83 to −2.09) at month 6, and −2.73 (95% CI −5.87 to 0.41) at month 12.

##### Long-Term Efficacy—Young Mania Rating Scale (YMRS)

For YMRS, no significant intervention effect was observed at any long-term treatment visit: V3, F(1,84) = 0.07, *p* = 0.794; V4, F(1,75) = 0.01, *p* = 0.914; and V5, F(1,60) = 0.75, *p* = 0.389. Baseline YMRS was not significantly associated with the outcome at any of these visits.

##### Long-Term Efficacy—Clinical Global Impression (CGI)

For CGI, no significant intervention effect was observed at V3, F(1,84) = 2.34, *p* = 0.130; V4, F(1,75) = 2.41, *p* = 0.125; or V5, F(1,60) = 1.73, *p* = 0.193. Baseline CGI was also not significantly associated with the outcome at any of these visits.

##### Long-Term Efficacy—Global Assessment of Functioning (GAF)

For GAF, the intervention effect was not significant at V3, F(1,84) = 2.12, *p* = 0.149, although baseline GAF was significantly associated with the V3 score, F(1,84) = 14.10, *p* < 0.001. At V4, GAF was significantly higher in the ASA group, F(1,75) = 11.51, *p* = 0.001, with adjusted mean scores of 7.40 versus 6.37; baseline GAF was not significant (*p* = 0.156). At V5, no significant intervention effect was observed, F(1,60) = 0.05, *p* = 0.831.

##### Response and Remission Rates During the Treatment Period

At the long-term treatment visits, response and depressive remission were compared between groups using Pearson χ^2^ tests without continuity correction, with Fisher’s exact tests as robustness checks. At V3, response was achieved by 81.4% of participants receiving ASA compared with 56.8% receiving placebo (χ^2^ = 6.14, *p* = 0.013; Fisher’s exact *p* = 0.020), whereas depressive remission rates did not differ significantly (67.4% vs. 52.3%, χ^2^ = 2.08, *p* = 0.149; Fisher’s exact *p* = 0.191). At V4, both response (89.5% vs. 50.0%, χ^2^ = 14.25, *p* < 0.001; Fisher’s exact *p* < 0.001) and remission rates (84.2% vs. 50.0%, χ^2^ = 10.26, *p* = 0.001; Fisher’s exact *p* = 0.002) were significantly more frequent in the ASA group. At V5, response rates were 81.8% versus 60.0% (χ^2^ = 3.67, *p* = 0.056; Fisher’s exact *p* = 0.093), while depressive remission was observed in 81.8% versus 56.7% (χ^2^ = 4.72, *p* = 0.030; Fisher’s exact *p* = 0.053). Per-visit response and remission results are summarised in Table 2.

##### Bipolar Remission During the Treatment Period

Bipolar remission, defined as HAMD-17 ≤ 7 and YMRS ≤ 7, was more frequent in the ASA group at each long-term treatment visit. At V3, remission rates were 65.1% with ASA and 52.3% with placebo (χ^2^ = 1.48, *p* = 0.224; Fisher’s exact *p* = 0.278). A significant between-group difference was observed at V4 (81.6% vs. 50.0%, χ^2^ = 8.59, *p* = 0.003; Fisher’s exact *p* = 0.004). At V5, remission rates were 75.8% versus 56.7%, respectively (χ^2^ = 2.58, *p* = 0.108; Fisher’s exact *p* = 0.120).

Overall, the categorical efficacy outcomes showed the clearest separation between groups at V4 (month 6), with significantly higher response, depressive remission, and bipolar remission rates in the ASA group.

##### Suicide Attempts During the Study Period

No suicide attempts were reported in either treatment group during the entire study period.

### 3.5. Adherence

Medication adherence assessed using the Medication Adherence Rating Scale (MARS) remained high throughout the study and was comparable between treatment groups. No significant treatment-by-visit interaction was observed (*p* = 0.502). Mean MARS scores remained approximately 8 points in both groups across the available assessments.

The proportion of participants meeting the pre-specified adherence threshold of ≥80% by pill count was 45/47 (95.7%) in the ASA group and 46/48 (95.8%) in the placebo group.

### 3.6. Safety

Safety outcomes were comparable between the ASA and placebo groups during both the 12-month treatment period and the one-month post-treatment period. During the 12-month treatment period, adverse events were reported in 8/50 (16.0%) participants receiving ASA and 7/50 (14.0%) receiving placebo, while one serious adverse event occurred in the ASA group and none in the placebo group (psychiatric hospitalisation due to worsening depressive symptoms). Two distinct psychiatric hospitalisations occurred in the ASA group. One was associated with worsening depressive symptoms and suicidal ideation, and the second with a manic episode. No clinically reported hypomanic episodes were documented. A descriptive review of the eCRF adverse-event records identified 19 initial AE reports in the ASA group and 13 in the placebo group, once follow-up reports referring to the same event were not counted as separate events. In the ASA group, 16 events were classified as mild, two as moderate, and one as severe; in the placebo group, 12 were mild and one was moderate, with no events classified as severe. Most recorded events were isolated laboratory or ECG abnormalities (10 in the ASA group and 11 in the placebo group), while the remaining reports comprised intercurrent infections or other somatic symptoms and psychiatric events. Protocol-defined laboratory, coagulation, and ECG abnormalities are reported separately in Table 3.

No adverse events or serious adverse events were reported during the post-treatment period between months 12 and 13. No statistically significant between-group differences were observed in clinically relevant vital-sign abnormalities, body-weight changes, ECG abnormalities, coagulation parameters, bleeding time, or overall laboratory abnormalities during either period. Detailed safety outcomes are presented in Table 3.

### 3.7. Exploratory Tertiary Analysis: Somatic Comorbidity

Exploratory analyses suggested a greater treatment benefit of ASA among participants with somatic comorbidities, particularly at later visits. Within this subgroup, baseline-adjusted HAMD-17 differences favoured ASA at V4 (−7.06 points, 95% CI −11.60 to −2.51, *p* = 0.003) and V6 (−7.59 points, 95% CI −13.39 to −1.79, *p* = 0.011). At V4, both clinical response and depressive remission were observed in 87.5% of ASA-treated participants with somatic comorbidity compared with 33.3% of placebo-treated participants (Fisher’s exact *p* = 0.003 for both). At V6, depressive remission was observed in 91.7% versus 22.2%, respectively (Fisher’s exact *p* = 0.002). However, formal treatment-by-comorbidity interaction tests did not reach statistical significance (HAMD-17 at V6, *p* for interaction = 0.078; remission at V6, *p* for interaction = 0.068); therefore, these findings should be considered exploratory.

## 4. Discussion

### 4.1. Principal Findings

In this multicentre, randomised, double-blind, placebo-controlled phase II trial, adjunctive acetylsalicylic acid (ASA) 150 mg/day produced a clinically meaningful antidepressant effect in patients with bipolar depression after 2 months of treatment and was associated with a significantly lower rate of relapse or clinical worsening during 12 months of treatment. The magnitude of the short-term effect on depressive symptoms was within the range reported in trials of established pharmacotherapies for bipolar I depression, including lurasidone, and cariprazine [6,26,27,28], and within the pooled effect estimate reported for adjunctive anti-inflammatory agents in bipolar depression [16]. For clinical context, the NNT for the composite long-term endpoint was approximately 5; however, this estimate is not directly comparable with NNTs derived from acute response or remission outcomes in trials of established treatments [26] because of differences in endpoint definition, treatment duration, and study populations. These cross-trial comparisons are intended only to provide clinical context and should be interpreted cautiously because the studies differed in patient populations, outcome definitions, treatment duration, and follow-up schedules. The composite endpoint incorporated clinically meaningful events—affective hospitalisation, intensification of pharmacotherapy, suicidality, and marked clinical worsening on the CGI—and its reduction is potentially important because relapse and clinical worsening are central determinants of disability and premature mortality in bipolar disorder [2,4].

Secondary efficacy outcomes were broadly consistent with the primary findings. Response and remission rates were numerically higher with ASA at month 2 but did not reach conventional statistical significance. The largest numerical separation between groups in the visit-specific analyses was observed at month 6, when response, depressive remission, and bipolar remission (defined by both HAMD-17 and YMRS ≤ 7) were all significantly more frequent in the ASA group. Notably, month 6 represented the point of greatest convergence across efficacy domains: ASA was associated with a significant reduction in depressive symptom severity on HAMD-17, higher rates of clinical response, depressive remission, and bipolar remission, and significantly better overall functioning on the GAF. The higher bipolar remission rate indicates that the antidepressant benefit was achieved without a corresponding increase in manic symptoms—an important safety consideration in bipolar depression, where mania limits the utility of several conventional antidepressant strategies [6,7].

### 4.2. Timing of the Treatment Effect

The visit-specific findings showed the largest numerical differences between groups at month 6; however, given the exploratory nature of these secondary analyses, the number of comparisons, and the smaller samples at later visits, this pattern should be interpreted cautiously. Anti-inflammatory agents in mood disorders have previously been observed to exert a delayed antidepressant effect, with some trials reporting maximal benefit only after weeks to months of exposure [16,29]. Several pharmacodynamic considerations may be relevant: low-dose ASA irreversibly acetylates cyclooxygenase-1 (COX-1) and, at chronic exposure, is thought to trigger COX-2-dependent production of specialised pro-resolving mediators (SPMs), including 15-epi-lipoxin A4 [30]. SPMs and downstream lipid mediators accumulate progressively during continued daily dosing and are considered central to the resolution phase of inflammation rather than to its acute suppression. Chronic low-dose ASA may also modulate microglial activation and downregulate NF-κB signalling in the central nervous system [31,32], and animal work suggests stimulatory effects on oligodendrocyte-mediated myelination [31]. The GAF benefit observed at month 6 was not maintained at month 12 despite continued blinded treatment. This pattern should be interpreted cautiously, because the study was not designed to determine the optimal duration of ASA treatment or whether the magnitude of benefit changes over extended maintenance.

### 4.3. Findings in the Context of Previous Evidence

The present findings extend a limited and heterogeneous evidence base on anti-inflammatory strategies in bipolar depression. Earlier studies of ASA have generally been smaller, shorter, factorial in design, or focused on outcomes different from clinician-rated bipolar depressive symptoms. In the factorial trial by Savitz et al. [23], 99 outpatients received minocycline, aspirin (81 mg twice daily), both, or double placebo for 6 weeks; aspirin exposure was associated with a favourable sustained-response signal (OR = 3.7; *p* = 0.019; NNT = 4.0), although the main effect on the primary continuous depression outcome was not statistically significant. The smaller factorial trial by Bauer et al. [24] provided mixed evidence for aspirin-containing strategies at higher doses. More recently, the A-Bipolar RCT [25] enrolled 250 patients newly diagnosed with bipolar disorder and used the same daily ASA dose of 150 mg over 6 months but assessed daily self-reported mood instability (RMSSD) rather than clinician-rated affective symptoms; no benefit was found on the primary outcome or on secondary outcomes including a 6-item HAM-D. Several features distinguish A-Bipolar from ASPIRIN BD and may be relevant to the discordant findings: A-Bipolar recruited newly diagnosed, largely euthymic patients rather than participants with an active depressive episode, and its primary endpoint quantified short-term mood variability rather than depressive symptom burden or clinically meaningful relapse. The 2026 randomised study of aspirin (100 mg/day) and atorvastatin (10 mg/day) as augmentation in affective disorders with inflammatory dysregulation (CRP ≥ 1000 ng/mL or TNF-αR1 ≥ 800 pg/mL) reported significant reductions in HDRS and MADRS scores in both active-treatment groups compared with placebo (both *p* < 0.001), providing additional evidence that ASA may have antidepressant activity in patients selected for inflammatory dysregulation [33]. Taken together, ASPIRIN BD addresses a distinct clinical question—adjunctive ASA in an acute bipolar depressive episode using clinician-rated depressive symptoms as the primary outcome—and its findings are complementary to, rather than in conflict with, the A-Bipolar RCT.

The results should also be considered in the broader context of trials of anti-inflammatory treatments in mood disorders. Findings with celecoxib, minocycline, N-acetylcysteine (NAC), and other anti-inflammatory or immunomodulatory approaches have been inconsistent. The MINDCARE trial [20] did not confirm superiority of celecoxib or minocycline over placebo for the primary depression outcome in bipolar depression in a lower-middle-income-country setting, and a subsequent meta-analysis of NAC augmentation in bipolar depression reported a moderate effect (Cohen’s d = 0.45; 95% CI 0.06 to 0.84) with substantial heterogeneity [34]. Adjunctive anti-inflammatory strategies as a class have shown pooled but heterogeneous benefit [16,18,35], and pharmacoepidemiological data from Danish nationwide registers demonstrated that continued low-dose ASA use was associated with a reduced incidence of mania/bipolar disorder in over 1.6 million individuals—an effect not observed with non-aspirin NSAIDs or high-dose ASA [22]. This class heterogeneity may reflect differences in patient populations, illness stage, concomitant treatment, biological characteristics, and the pharmacological mechanisms of the agents studied. Low-dose ASA is pharmacologically distinctive, acting through irreversible acetylation of COX-1, dose-dependent modulation of COX-2, and downstream generation of pro-resolving lipid mediators, and its favourable safety and access profile differentiates it from most anti-inflammatory compounds evaluated to date. The current findings support further investigation of low-dose ASA as a potential adjunctive treatment for bipolar depression, while the extent to which these results generalise to other anti-inflammatory strategies remains uncertain.

### 4.4. Exploratory Role of Somatic Comorbidity

Exploratory analyses showed numerically larger between-group differences in participants with somatic comorbidities at selected later assessments, including HAMD-17 at month 6 and the post-treatment month-13 assessment, as well as higher remission rates at month 13 in the ASA-treated subgroup. However, formal treatment-by-comorbidity interaction tests did not reach statistical significance. These findings should therefore be regarded as hypothesis-generating and do not establish somatic comorbidity as a treatment-response moderator. Nevertheless, they are biologically plausible and consistent with several converging lines of evidence: low-grade inflammation, defined as CRP > 3 mg/L, has been reported in the majority of individuals with bipolar depression and is preferentially associated with anhedonia, fatigue, and psychomotor retardation [15]; patients with bipolar disorder have consistently elevated peripheral IL-6, TNF-α, and CRP compared with healthy controls, with the largest CRP elevations observed during mania but persistent elevations during depression and euthymia [36]; and bipolar disorder is associated with markedly excess cardiovascular and metabolic mortality [4]. In this context, the numerically larger treatment effects observed in participants with somatic comorbidity provide a rationale for prospective investigation of whether specific cardiometabolic, endocrine, immune/inflammatory, or other clinically defined phenotypes are associated with differential response to ASA. Somatic comorbidity itself should not be regarded as a surrogate for inflammatory activation. Recent meta-analytic evidence also highlights substantial heterogeneity in peripheral inflammatory biomarkers, supporting prospective biomarker-based stratification rather than reliance on clinical comorbidity alone [37]. Future prospective trials could test whether stratification by baseline CRP, IL-6, or other predefined inflammatory phenotype markers improves identification of patients who may benefit from ASA [15], building on stratification approaches previously explored with other anti-inflammatory agents [33,38,39]. It should be emphasised that the biomarker analyses planned within the ASPIRIN BD trial—including baseline and longitudinal CRP and IL-6—are ongoing and will be reported separately; they represent the primary means by which the mechanistic hypotheses generated here can be tested empirically. More broadly, heterogeneity in bipolar disorder may also reflect non-inflammatory biological factors. For example, exploratory data on PER3 VNTR variation have suggested associations with age at onset and depressive-episode burden in bipolar I disorder, supporting the broader concept that circadian-genetic as well as inflammatory and clinical characteristics may contribute to illness heterogeneity [40].

### 4.5. Potential Mechanisms

The biological mechanism underlying the observed clinical effect cannot be established from the present analyses, and any mechanistic interpretation remains necessarily hypothetical. At a dose of 150 mg/day, ASA is expected to act predominantly through platelet COX-1 inhibition rather than through the broad systemic anti-inflammatory effects associated with much higher doses. Nevertheless, this does not exclude more subtle peripheral immunomodulatory effects, including effects mediated through platelet–immune interactions and lipid-derived inflammatory or pro-resolving pathways. At the same time, a central contribution is also biologically plausible, as ASA and its principal metabolite salicylate can cross the blood–brain barrier and may influence neuroinflammatory signalling within the central nervous system. The observed clinical effect may therefore reflect central mechanisms, peripheral immunomodulatory effects, or an interaction between the two. Inter-individual differences in blood–brain barrier integrity, transporter activity, and metabolic–vascular comorbidity could further contribute to heterogeneity in central drug exposure and clinical response in ways that are not fully captured by peripheral biomarkers.

Several central mechanisms with converging preclinical and clinical support are relevant. First, low-dose ASA irreversibly inhibits COX-1 in platelets and, over sustained exposure, produces a dose-dependent modulation of COX-2 activity in peripheral and central tissues [31]. COX-2-derived prostaglandin E2 has been implicated in stress-induced affective and cognitive changes in preclinical models [41]. Second, ASA-triggered lipoxin generation and downstream specialised pro-resolving mediators (SPMs) may re-establish inflammatory resolution rather than simply blocking inflammation initiation [30], a mechanism that is pharmacodynamically distinct from that of most other anti-inflammatory agents. Its relevance to the clinical time course observed in the present trial remains speculative. Third, preclinical studies have suggested interactions between ASA and lithium involving brain COX-2 and prostaglandin metabolism, together with possible effects of low-dose ASA on oligodendrocyte-mediated myelination [31]. However, these observations cannot be directly extrapolated to the present trial, particularly because lithium was used by only 24% of participants in the ASA group and 22% in the placebo group. Fourth, chronic systemic inflammation shifts tryptophan metabolism from serotonin toward the kynurenine pathway, generating neuroactive metabolites with plausible relevance to depressive symptomatology, glutamatergic dysregulation, and cognitive dysfunction [32,42]. Through these inflammatory and kynurenine-related pathways, ASA could indirectly influence serotonergic and glutamatergic neurotransmission. However, a direct effect of low-dose ASA on brain neurotransmitter concentrations has not been established, and neurotransmitter levels were not measured in the present study. Fifth, glial and microglial changes have been reported in bipolar disorder—including elevated TSPO binding in the hippocampus of euthymic BD-I patients and post-mortem microglial changes in a subset of BD cases [32]—and low-dose ASA may modulate microglial activation and NF-κB signalling. Together, these observations provide a biological rationale for further mechanistic investigation of low-dose ASA in bipolar depression. However, none of these pathways was directly assessed in the present clinical analyses, and they should therefore be regarded as candidate mechanisms rather than established explanations for the observed treatment effect.

### 4.6. Safety and Adherence

Medication adherence was high and comparable between groups, making differential treatment exposure an unlikely explanation for the observed efficacy differences. ASA was generally well tolerated. No statistically significant between-group differences were observed in adverse events, serious adverse events, or protocol-defined clinical and laboratory safety outcomes during the 12-month treatment period or during the one-month post-treatment follow-up. In particular, no excess of abnormalities in vital signs, clinically relevant body-weight change, ECG findings, coagulation parameters, bleeding time, or overall laboratory safety measures was observed among participants receiving ASA. Importantly, no clinically evident bleeding events were observed, including gastrointestinal bleeding, excessive menstrual bleeding, or other bleeding manifestations suggestive of increased haemorrhagic risk. Protocol-defined coagulation abnormalities were observed in both groups, but none was associated with a clinically evident bleeding event or other clinically consequential coagulation complication. Notably, no bleeding events were reported among participants receiving concomitant SSRI treatment. These findings support the tolerability of adjunctive low-dose ASA in this selected study population, although the sample size and incomplete later assessments were insufficient to exclude uncommon adverse effects, including bleeding complications.

Several safety considerations are important for interpreting these reassuring findings. First, the trial deliberately enrolled a population at low a priori bleeding risk: patients with a history of peptic ulceration, gastrointestinal bleeding, or any known bleeding diathesis were excluded per protocol, as were patients receiving anticoagulants, antiplatelet agents, or chronic NSAIDs. Second, the trial recruited a comparatively young population (age 18–60 years); risk–benefit considerations differ substantially in older adults. The 2022 U.S. Preventive Services Task Force recommendation on aspirin for primary cardiovascular prevention explicitly recommends against initiating low-dose aspirin in adults aged 60 years or older (Grade D), on the basis of a shift in the risk–benefit balance driven by rising rates of major bleeding—including gastrointestinal and intracranial haemorrhage—with age [43]. Any extension of the present findings to older patients with bipolar depression would therefore require independent evaluation of safety in that population. Third, chronic low-dose ASA carries an increased risk of upper gastrointestinal bleeding when co-administered with selective serotonin reuptake inhibitors [44]; gastroprotective co-therapy (e.g., proton-pump inhibitors) should be considered when ASA is combined with an SSRI in future pragmatic use. It is worth emphasising, in this context, that a large meta-analysis of observational data reporting a small increase in incident depression with aspirin use (OR 1.10) included subgroup associations for aspirin exposure lasting ≥ 5 years and at doses ≥ 500 mg/day; these exposure patterns differ substantially from the 150 mg/day regimen evaluated in the present trial [45]. Finally, the protocol-mandated exclusion of participants receiving valproate—necessary because of the pharmacokinetic interaction between valproate and salicylates—limits the applicability of the safety findings to that subgroup, which would require separate evaluation.

### 4.7. Strengths and Limitations

Strengths include the multicentre randomised design, a clinician-rated primary outcome, recruitment of participants experiencing a bipolar depressive episode, and a 12-month treatment period. The trial combined clinician-rated symptom outcomes with response, remission, functional measures, a clinically meaningful long-term composite endpoint, frequent safety monitoring, and adherence assessments. The consistency between the primary short-term analysis and the long-term primary endpoint further supports the robustness of the principal findings. The trial was prospectively registered in EudraCT and subsequently transitioned to CTIS.

Several limitations should also be considered. First, only one ASA dose (150 mg/day) was tested, precluding conclusions about dose–response relationships. This dose was selected on the basis of observational and pharmacoepidemiological evidence associating low-dose ASA with reduced affective episode rates [21,22], but the possibility that a higher dose or an alternative dosing schedule would produce a larger or a smaller effect cannot be excluded. Second, several a priori eligibility restrictions limit the direct generalisability of the findings: participants receiving valproate were excluded per protocol because of the pharmacokinetic interaction with salicylates; participants with a history of peptic ulceration, gastrointestinal bleeding, or any bleeding diathesis were excluded on safety grounds; and enrolment was restricted to adults aged 18–60 years, so the safety and efficacy of adjunctive low-dose ASA in older patients with bipolar depression—in whom bleeding risk is substantially higher [43]—cannot be inferred from these data. Third, attrition increased during later visits, substantially reducing the number of observed cases at months 6 and 12. Although the long-term primary analysis addressed missing data conservatively using non-responder imputation and was supported by a complete-case sensitivity analysis, attrition reduces precision for secondary outcomes at later visits. Fourth, the study was conducted exclusively in Poland and predominantly recruited outpatients from four psychiatric centres; replication in more diverse populations, healthcare settings, and health-system contexts is required to establish generalisability. Fifth, multiple secondary and exploratory comparisons were performed; isolated significant findings, particularly subgroup results, should therefore be interpreted cautiously. Sixth, GAF was analysed using coded ordinal categories rather than the original 0–100 scale, which may reduce sensitivity and complicate interpretation of small between-group differences. Seventh, the long-term primary endpoint was a composite of clinically heterogeneous events (hospitalisation, pharmacotherapy intensification, suicidality, marked CGI worsening), and the trial was not powered to examine its individual components separately. Blinding integrity was not formally assessed using a structured post-trial evaluation, although the placebo tablet was matched to ASA in appearance, taste, and smell. Finally, biomarker analyses of CRP and IL-6, which are essential for interpreting the mechanistic significance of the clinical findings and for defining candidate response predictors, are ongoing and will be reported in a subsequent paper.

### 4.8. Clinical Implications and Future Research

Current CANMAT/ISBD recommendations provide the established pharmacological treatment framework for bipolar depression [46], while ongoing clinical research continues to evaluate new adjunctive strategies for patients with persistent unmet therapeutic needs. The present findings suggest that low-dose ASA may represent one such investigational option. ASA, an inexpensive and widely available agent with a well-characterised safety profile, has clinically meaningful antidepressant activity in bipolar depression and may reduce the rate of relapse or clinical worsening during 12 months of adjunctive treatment. Rather than requiring direct replication alone, the trial may be viewed as a proof of concept that opens two complementary lines of subsequent research. First, and most important, is the identification of the subgroup of patients most likely to benefit. As with other established treatments in bipolar disorder, the clinical value of ASA may ultimately depend on identifying patients with a higher probability of response. Priority candidate enrichment strategies include stratification by baseline inflammatory phenotype (CRP > 3 mg/L; IL-6; composite inflammatory scores) and by defined somatic comorbidity profiles (metabolic syndrome, cardiovascular disease, chronic inflammatory conditions). Given the partially overlapping anti-inflammatory and prostaglandin-modulating pathways implicated in lithium and low-dose ASA, future studies might also explore whether clinical or biological features associated with lithium responsiveness are relevant to ASA response, although this was not examined in the present trial [31]. Prospective biomarker-stratified designs that pre-specify these subgroups would be considerably more informative than an undifferentiated replication attempt. Recent reviews of psychiatric immunomodulatory trials similarly emphasise biomarker enrichment, phenotype selection, and careful long-term safety assessment as priorities for future studies [47]. Second, mechanistic sub-studies incorporating platelet reactivity, prostaglandin metabolites, specialised pro-resolving mediators, kynurenine-pathway metabolites, and—where feasible—TSPO PET or other markers of central microglial activation [30,32,42] would help clarify whether the clinical effect is mediated predominantly through central mechanisms, peripheral immunomodulatory pathways, or an interaction between the two.

Additional priorities include: dose–response studies to determine whether 150 mg/day represents the optimal balance between efficacy and safety, and whether alternative low-dose regimens (e.g., 75–100 mg/day or intermittent dosing) confer comparable benefit with lower bleeding risk; pragmatic trials evaluating ASA against, or in combination with, currently first-line agents for bipolar depression [6,27,28]; trials specifically evaluating ASA in patients with somatic comorbidity, in whom both the anti-inflammatory and the cardioprotective actions of ASA may offer additive benefit; longer-term maintenance studies to determine whether the clinical benefit observed at 6 months is maintained beyond 12 months; and dedicated evaluation in older adults, in whom the bleeding-risk profile is materially different [43] and separate risk–benefit assessment is required. Given the excess cardiovascular and metabolic mortality in bipolar disorder [4], future trials should also be designed to capture cardiovascular outcomes in parallel with psychiatric outcomes.

## 5. Conclusions

In conclusion, adjunctive ASA 150 mg/day produced a clinically meaningful reduction in depressive symptoms at the pre-specified short-term primary endpoint and was associated with a significantly lower rate of relapse or clinical worsening during 12 months of treatment. The magnitude of the short-term antidepressant effect was within the range reported for established pharmacotherapies for bipolar depression, and the long-term composite endpoint yielded an NNT of 5. Secondary outcomes provided additional evidence of benefit at selected later visits, particularly at month 6, without evidence of increased manic symptoms or a clinically relevant safety signal. These findings support the further investigation of low-dose ASA as an adjunctive treatment for bipolar depression, with priority given to biomarker-stratified and phenotype-enriched studies that identify the patients most likely to benefit, and to mechanistic sub-studies that test the hypothesised central, resolution-promoting action of low-dose ASA in mood disorders.

## Figures and Tables

**Table 1:** Baseline demographic, clinical, and laboratory characteristics of the ITT population by treatment arm.

Variable	ASA (n = 50)	Placebo (n = 50)	*p* Value	SMD
Demographics				
Age, years, mean (SD)	39.18 (11.50)	38.82 (10.72)	0.872	+0.032
Sex, n (%)			0.837	+0.082
Female	32 (64.0%)	30 (60.0%)		
Male	18 (36.0%)	20 (40.0%)		
Education, n (%)			0.264	—
Higher	32 (64.0%)	26 (52.0%)		
Secondary	17 (34.0%)	20 (40.0%)		
Primary	1 (2.0%)	4 (8.0%)		
Employment, n (%)			1.000	+0.000
Employed	33 (66.0%)	33 (66.0%)		
Not employed	17 (34.0%)	17 (34.0%)		
Place of residence, n (%)			0.126	—
Large city	38 (76.0%)	34 (68.0%)		
Medium-sized city	3 (6.0%)	5 (10.0%)		
Small town	6 (12.0%)	2 (4.0%)		
Village	3 (6.0%)	9 (18.0%)		
Recruiting site, n (%)			0.230	—
01 IPiN Warsaw	29 (58.0%)	23 (46.0%)		
02 Nowowiejski	12 (24.0%)	13 (26.0%)		
03 Świecie	1 (2.0%)	6 (12.0%)		
04 Holimedyk	8 (16.0%)	8 (16.0%)		
BD characteristics				
Bipolar disorder type, n (%)			0.202	+0.299
Type I	37 (74.0%)	30 (60.0%)		
Type II	13 (26.0%)	20 (40.0%)		
Age at BD onset, years, median (IQR)	21.5 (18.0–29.0)	24.0 (20.0–31.0)	0.385	−0.068
Illness duration, years, median (IQR)	14.0 (7.2–20.0)	13.0 (8.0–19.0)	0.913	+0.097
Prior hospitalisations, n, median (IQR)	1.5 (0.0–3.0)	1.0 (0.0–3.8)	0.501	+0.210
History of any suicide attempt, n (%)	14 (28.0%)	11 (22.0%)	0.644	+0.139
Prior suicide attempts, n, median (IQR)	0.0 (0.0–1.0)	0.0 (0.0–0.0)	0.586	+0.187
Family history of BD, n (%)	19 (38.0%)	16 (32.0%)	0.675	+0.126
Baseline psychopathology (V1)				
HAMD-17, mean (SD)	15.92 (6.91)	16.70 (5.88)	0.545	−0.122
YMRS, mean (SD)	1.44 (2.18)	1.60 (1.63)	0.678	−0.083
CGI-Severity, mean (SD)	4.06 (1.11)	3.98 (0.91)	0.696	+0.078
GAF, coded category, mean (SD)	6.70 (1.39)	6.60 (1.05)	0.685	+0.081
Concomitant psychiatric medications at baseline				
Lithium, n (%)	12 (24.0%)	11 (22.0%)	1.000	+0.048
Lamotrigine, n (%)	32 (64.0%)	36 (72.0%)	0.520	−0.172
Carbamazepine, n (%)	2 (4.0%)	6 (12.0%)	0.269	−0.305
Valproate, n (%)	0 (0.0%)	0 (0.0%)	1.000	0.000
Quetiapine, n (%)	20 (40.0%)	19 (38.0%)	1.000	+0.041
Olanzapine, n (%)	5 (10.0%)	2 (4.0%)	0.436	+0.241
Aripiprazole, n (%)	15 (30.0%)	18 (36.0%)	0.671	−0.128
Any SGA, n (%)	36 (72.0%)	30 (60.0%)	0.291	+0.254
Any antidepressant, n (%)	25 (50.0%)	28 (56.0%)	0.689	−0.120
Somatic comorbidities				
Any somatic comorbidity, n (%)	21 (42.0%)	18 (36.0%)	0.682	+0.123
Diabetes/insulin resistance, n (%)	8 (16.0%)	6 (12.0%)	0.773	+0.116
Hypertension, n (%)	5 (10.0%)	4 (8.0%)	1.000	+0.070
Thyroid disease, n (%)	7 (14.0%)	4 (8.0%)	0.523	+0.193
Obesity/overweight (self-reported), n (%)	3 (6.0%)	1 (2.0%)	0.617	+0.211
Dyslipidaemia, n (%)	1 (2.0%)	1 (2.0%)	1.000	+0.000
Cardiovascular disease, n (%)	2 (4.0%)	0 (0.0%)	0.495	+0.403
Gastrointestinal disease, n (%)	2 (4.0%)	3 (6.0%)	1.000	−0.092
Baseline laboratory (V1)				
Weight, kg, median (IQR)	77.5 (65.0–87.8)	78.5 (67.8–94.5)	0.456	−0.129
Heart rate, bpm, median (IQR)	72.0 (70.0–80.0)	72.5 (70.0–80.0)	0.994	−0.037
WBC, ×10^9^/L, mean (SD)	6.53 (1.84)	6.69 (2.37)	0.687	−0.076
RBC, ×10^12^/L, mean (SD)	4.63 (0.50)	4.72 (0.52)	0.380	−0.180
Haemoglobin, g/dL, mean (SD)	13.80 (1.87)	14.20 (1.51)	0.431	−0.232
Platelets, ×10^9^/L, mean (SD)	264.14 (57.58)	261.51 (69.83)	0.841	+0.041
CRP, mg/L, mean (SD)	1.87 (2.62)	3.34 (7.98)	0.719	−0.246
IL-6, pg/mL, mean (SD)	2.79 (2.41)	3.53 (4.11)	0.663	−0.220
Total cholesterol, mg/dL, mean (SD)	196.96 (43.87)	190.29 (30.68)	0.390	+0.176
LDL, mg/dL, mean (SD)	125.24 (40.97)	120.40 (28.78)	0.976	+0.136
HDL, mg/dL, mean (SD)	58.91 (21.08)	58.00 (27.17)	0.308	+0.038
Triglycerides, mg/dL, mean (SD)	124.49 (77.18)	134.09 (84.91)	0.479	−0.118
TSH, mIU/L, mean (SD)	1.97 (1.12)	1.84 (0.99)	0.657	+0.126
D-dimer, ng/mL, mean (SD)	332.02 (193.48)	282.83 (191.76)	0.073	+0.255
INR, mean (SD)	1.02 (0.11)	1.01 (0.09)	0.916	+0.085

Notes: ASA, acetylsalicylic acid (150 mg/day); BD, bipolar disorder; CGI, Clinical Global Impression; CRP, C-reactive protein; GAF, Global Assessment of Functioning; HAMD-17, 17-item Hamilton Depression Rating Scale; HDL, high-density lipoprotein; IL-6, interleukin-6; INR, international normalised ratio; ITT, intention-to-treat; LDL, low-density lipoprotein; SGA, second-generation antipsychotic; SMD, standardised mean difference; TSH, thyroid-stimulating hormone; WBC, white blood cell count; YMRS, Young Mania Rating Scale. Baseline laboratory variables are presented as mean (SD); other continuous variables are presented as mean (SD) or median (IQR), as appropriate; categorical variables as n (%). *p* values are unadjusted and are provided descriptively; they were not used to assess the success of randomisation. SMDs are provided as an additional measure of baseline balance. Data note: GAF was recorded in 10-point ranges and converted to ordered numerical categories according to the prespecified coding scheme. Laboratory results recorded as not done were treated as missing.

**Table 2:** Efficacy outcomes in the ASPIRIN BD trial.

Outcome/Visit	ASA	Placebo	Between-Group Effect	*p* Value
Short-term primary endpoint: HAMD-17 at month 2				
V2 primary analysis, mITT observed cases	8.13 (n = 47)	12.37 (n = 48)	Adjusted difference −4.24 [−7.20, −1.28]	**0.005**
V2 sensitivity analysis, ITT with LOCF	Δ −7.38 (SD 6.31)	Δ −3.72 (SD 8.88)	Adjusted difference −3.97 [−6.88, −1.06]	**0.008**
Long-term primary endpoint: relapse or clinical worsening during months 4–12				
Primary analysis, ITT with NRI	31/50 (62.0%)	41/50 (82.0%)	OR 0.36 (95% CI 0.14–0.90); ARR 20.0 pp (95% CI 2.8–37.2); NNT 5	**0.026**
Sensitivity analysis, complete cases	14/33 (42.4%)	22/31 (71.0%)	OR 0.30; ARR 28.6 pp; NNT ≈4	**0.021**
Long-term HAMD-17 outcomes (ANCOVA, baseline-adjusted)				
V3 (month 4)	6.28 (n = 43)	8.80 (n = 44)	Adjusted difference −2.52 [−5.22, 0.18]	0.067
V4 (month 6)	4.69 (n = 38)	9.69 (n = 40)	Adjusted difference −5.00 [−7.86, −2.13]	**<0.001**
V5 (month 12)	4.73 (n = 33)	7.60 (n = 30)	Adjusted difference −2.87 [−6.06, 0.32]	0.077
Clinical response (≥50% reduction in HAMD-17 from baseline)				
V2 (month 2)	28/47 (59.6%)	20/48 (41.7%)	Pearson χ^2^ = 3.05	0.081
V3 (month 4)	35/43 (81.4%)	25/44 (56.8%)	Pearson χ^2^ = 6.14	**0.013**
V4 (month 6)	34/38 (89.5%)	20/40 (50.0%)	Pearson χ^2^ = 14.25	**<0.001**
V5 (month 12)	27/33 (81.8%)	18/30 (60.0%)	Pearson χ^2^ = 3.67	0.056
V6 (month 13, post-treatment)	22/28 (78.6%)	16/29 (55.2%)	Pearson χ^2^ = 3.51	0.061
Depressive remission (HAMD-17 ≤ 7)				
V2 (month 2)	27/47 (57.4%)	18/48 (37.5%)	Pearson χ^2^ = 3.79	0.052
V3 (month 4)	29/43 (67.4%)	23/44 (52.3%)	Pearson χ^2^ = 2.08	0.149
V4 (month 6)	32/38 (84.2%)	20/40 (50.0%)	Pearson χ^2^ = 10.26	**0.001**
V5 (month 12)	27/33 (81.8%)	17/30 (56.7%)	Pearson χ^2^ = 4.72	**0.030**
V6 (month 13, post-treatment)	23/28 (82.1%)	14/29 (48.3%)	Pearson χ^2^ = 7.17	**0.007**
Bipolar remission (HAMD-17 ≤ 7 and YMRS ≤ 7)				
V2 (month 2)	27/47 (57.4%)	18/48 (37.5%)	Pearson χ^2^ = 3.79	0.052
V3 (month 4)	28/43 (65.1%)	23/44 (52.3%)	Pearson χ^2^ = 1.48	0.224
V4 (month 6)	31/38 (81.6%)	20/40 (50.0%)	Pearson χ^2^ = 8.59	**0.003**
V5 (month 12)	25/33 (75.8%)	17/30 (56.7%)	Pearson χ^2^ = 2.58	0.108
V6 (month 13, post-treatment)	23/28 (82.1%)	14/29 (48.3%)	Pearson χ^2^ = 7.17	**0.007**
Other secondary continuous outcomes (baseline-adjusted ANCOVA)				
YMRS V2	1.32	1.12	F(1,92) = 0.09	0.770
YMRS V3	1.19	1.34	F(1,84) = 0.07	0.794
YMRS V4	0.81	0.86	F(1,75) = 0.01	0.914
YMRS V5	1.44	0.85	F(1,60) = 0.75	0.389
CGI V2	3.20	3.40	F(1,92) = 0.45	0.504
CGI V3	3.38	3.81	F(1,84) = 2.34	0.130
CGI V4	3.34	3.80	F(1,75) = 2.41	0.125
CGI V5	3.11	3.58	F(1,60) = 1.73	0.193
GAF V2 (coded)	6.56	6.12	F(1,92) = 1.70	0.195
GAF V3 (coded)	6.88	6.50	F(1,84) = 2.12	0.149
GAF V4 (coded)	7.40	6.37	F(1,75) = 11.51	**0.001**
GAF V5 (coded)	7.24	7.17	F(1,60) = 0.05	0.831

Values are adjusted means or n/N (%) unless otherwise stated. The V2 short-term primary analysis used ANCOVA in the mITT population with the baseline HAMD-17 score as a covariate; ITT with LOCF was the sensitivity analysis. The long-term primary endpoint used ITT with non-responder imputation (NRI), whereby missing long-term outcome data were classified as relapse/clinical worsening; a complete-case analysis was performed as a sensitivity analysis. Long-term continuous outcomes at V3–V5 were analysed separately by ANCOVA with the corresponding baseline score as a covariate. Categorical secondary outcomes were analysed in observed cases using Pearson’s χ^2^ test as the primary comparison; Fisher’s exact test was additionally used as a robustness check. For depressive remission at month 12, the two tests differed with respect to the conventional 0.05 significance threshold (Pearson *p* = 0.030; Fisher’s exact *p* = 0.053). V1 = baseline; V2 = month 2; V3 = month 4; V4 = month 6; V5 = month 12; V6 = month 13 post-treatment. ARR = absolute risk reduction; CGI = Clinical Global Impression; GAF = Global Assessment of Functioning; HAMD-17 = 17-item Hamilton Depression Rating Scale; ITT = intention-to-treat; LOCF = last observation carried forward; mITT = modified intention-to-treat; NNT = number needed to treat; OR = odds ratio; YMRS = Young Mania Rating Scale.

**Table 3:** Safety outcomes and treatment adherence.

Safety Outcome	ASA, n/N (%)	Placebo, n/N (%)	*p* Value
During the 12-month treatment period			
Any adverse event	8/50 (16.0%)	7/50 (14.0%)	0.790
Any serious adverse event	1/50 (2.0%)	0/50 (0.0%)	0.320
Clinically relevant change in vital signs	32/50 (64.0%)	32/50 (64.0%)	1.000
Body-weight change ≥ 7% from baseline at month 12	13/32 (40.6%)	10/29 (34.5%)	0.570
Protocol-defined ECG abnormality	7/46 (15.2%)	3/46 (6.5%)	0.180
Any abnormal coagulation parameter	19/48 (39.6%)	17/50 (34.0%)	0.550
Abnormal bleeding time	0/33 (0.0%)	2/34 (5.9%)	0.160
Any protocol-defined laboratory abnormality	28/46 (60.9%)	31/48 (64.6%)	0.590
During the post-treatment period (months 12–13)			
Any adverse event during months 12–13	0/30 (0.0%)	0/29 (0.0%)	1.000
Any serious adverse event during months 12–13	0/30 (0.0%)	0/29 (0.0%)	1.000
Clinically relevant change in vital signs during months 12–13	17/30 (56.7%)	11/29 (37.9%)	0.200
Body-weight change ≥ 7% from month 12 to month 13	0/25 (0.0%)	0/27 (0.0%)	1.000
Protocol-defined ECG abnormality at month 13	1/24 (4.2%)	1/27 (3.7%)	0.932
Any abnormal coagulation parameter at month 13	4/20 (20.0%)	9/26 (34.6%)	0.275
Abnormal bleeding time at month 13	0/12 (0.0%)	0/20 (0.0%)	1.000
Any protocol-defined laboratory abnormality at month 13	8/20 (40.0%)	14/26 (53.8%)	0.351
Treatment adherence			
Participants meeting ≥ 80% adherence by pill count	45/47 (95.7%)	46/48 (95.8%)	1.000
MARS treatment-by-visit interaction	—	—	0.502

Values are n/N (%) unless otherwise stated. Denominators reflect participants with evaluable data for the respective safety outcome and assessment period; no imputation was performed. Body-weight change at month 12 was defined as a change ≥ 7% from baseline, whereas post-treatment body-weight change was defined as a change ≥ 7% between months 12 and 13. Coagulation parameters included platelet count (PLT), D-dimer, international normalised ratio (INR), prothrombin time (PT), activated partial thromboplastin time (APTT), and thrombin time (TT). Protocol-defined laboratory abnormalities included all laboratory safety parameters specified in the study protocol, excluding coagulation parameters and bleeding time, which are reported separately. Between-group comparisons were performed using Pearson’s χ^2^ test without continuity correction, with Fisher’s exact test used as a robustness check, particularly for sparse data. AE = adverse event; APTT = activated partial thromboplastin time; ECG = electrocardiogram; INR = international normalised ratio; MARS = Medication Adherence Rating Scale; PLT = platelet count; PT = prothrombin time; SAE = serious adverse event; TT = thrombin time.

## Data Availability

The de-identified individual participant data underlying the findings reported in this article are available from the corresponding author on reasonable request, subject to an appropriate data-sharing agreement and applicable national and European data-protection regulations (including the General Data Protection Regulation, GDPR). The full study protocol (IPIN/ASPIRIN/BD, version 6.0, 28 February 2023), which includes the prespecified statistical analysis plan, is available from the corresponding author on reasonable request. Summary trial information is publicly accessible via EudraCT number 2021-006635-25 and EU CT number 2024-516584-86-00.

## Supplementary Materials

The following supporting information can be downloaded at: https://www.mdpi.com/article/10.3390/jcm15218349/s1. Table S1: CONSORT 2025 Checklist—ASPIRIN BD Trial.
