# Supplementary material for: Adjunctive Low-Dose Acetylsalicylic Acid in Bipolar Disorder: A Multicentre, Randomised, Double-Blind, Placebo-Controlled Phase II Trial (ASPIRIN BD)
Source: J Clin Med. 2026 Oct 27;15(21):8349. doi: 10.3390/jcm15218349 (PMC13763717; doi:10.3390/jcm15218349)
Supplement: Supplementary file 1 [file jcm-15-08349-s001.zip › jcm-4593144-supplementary.pdf]

CONSORT 2025 Checklist — ASPIRIN BD Trial

Adjunctive low-dose acetylsalicylic acid in bipolar depression: a multicentre, randomised, double-blind, placebo-controlled phase II trial

EudraCT 2021-006635-25 · EU CT 2024-516584-86-00 · Sponsor protocol IPIN/ASPIRIN/BD

This checklist maps the CONSORT 2025 reporting items to the current ASPIRIN BD manuscript. Locations are given by manuscript section/table/figure so they remain stable during final journal formatting. Items noted as not explicit or only partly reported should be considered during final pre-submission review.

Section / topic	Item	CONSORT 2025 reporting requirement	Reported in ASPIRIN BD manuscript / note
Title and abstract	1a	Identify the report as a randomised trial in the title.	Title: "Adjunctive low-dose acetylsalicylic acid in bipolar depression: a multicentre, randomised, double-blind, placebo-controlled phase II trial (ASPIRIN BD)".
Title and abstract	1b	Provide a structured summary of the trial design, methods, results, and conclusions.	Abstract: Background/Objectives, Methods, Results, Conclusions.
Open science – trial registration	2	Report the trial registry, registration identifier/URL, and registration date.	Methods – Trial design; Institutional Review Board Statement; Data Availability Statement. EudraCT 2021-006635-25 and EU CT 2024-516584-86-00 are reported; CTIS authorisation date (9 Aug 2024) is reported. The original EudraCT registration date is not stated explicitly.
Open science – protocol and SAP	3	State where the trial protocol and statistical analysis plan can be accessed.	Data Availability Statement: full protocol (version 6.0, 28 Feb 2023) and statistical analysis plan available from the corresponding author on reasonable request.
Open science – data sharing	4	State where and how de-identified participant-level data, statistical code, and other supporting materials can be accessed.	Data Availability Statement: de-identified participant-level data available from the corresponding author on reasonable request under a data-sharing agreement and applicable GDPR requirements; protocol and SAP also available. Statistical code is not separately specified.
Open science – funding / conflicts	5a	Report sources of funding or other support and the role of the funder(s).	Funding; Ethics, funding, and oversight: Polish Medical Research Agency (grant 2021/ABM/02/00008-00); funder role explicitly described.
Open science – funding / conflicts	5b	Report financial and other conflicts of interest of the manuscript authors.	Conflicts of Interest: authors declare no conflict of interest.
Introduction – background	6	Explain the scientific background and rationale for the trial.	Introduction: rationale for targeting inflammatory / immunomodulatory mechanisms in bipolar depression and for repurposing low-dose ASA.
Introduction – objectives	7	State specific objectives related to benefits and harms.	End of Introduction and Methods – Outcomes: short-term antidepressant efficacy, 12-month relapse/clinical worsening, and safety/tolerability.
Methods – patient/public involvement	8	Describe how patients or the public were involved in trial design, conduct, or reporting.	Methods – Patient and public involvement: patients/public were not research partners; recruitment information was disseminated through patient-facing websites and organisations.
Methods – trial design	9	Describe the trial design, allocation ratio, and trial framework.	Methods – Trial design: multicentre, randomised, double-blind, placebo-controlled, parallel-group phase II trial; 1:1 allocation. The manuscript does not explicitly label the framework as superiority/equivalence/non-inferiority.
Methods – changes after trial start	10	Describe important changes after trial commencement, including non-prespecified outcomes or analyses, with reasons.	Methods – Changes from protocol-specified statistical analyses: changes to primary/long-term analysis strategy are described; two non-prespecified interim safety reviews are also reported.
Methods – setting	11	Report the settings and locations where the trial was conducted.	Methods – Trial design / Participants: four psychiatric centres in Poland (Warsaw/IPiN, Nowowiejski Hospital, Świecie, Częstochowa/Holimedyk).
Methods – eligibility	12a	Report participant eligibility criteria.	Methods – Participants: detailed inclusion and exclusion criteria.
Methods – eligibility	12b	If applicable, report eligibility criteria for sites and for individuals delivering the intervention.	Not applicable as a procedural intervention; participating centres are named and study procedures were conducted by qualified clinical/research staff. No additional site/operator eligibility criteria are specified.
Methods – intervention/comparator	13	Describe intervention and comparator in enough detail to allow replication and indicate where supplementary intervention materials can be accessed, if relevant.	Methods – Interventions: enteric-coated ASA 150 mg once daily vs matching placebo for 12 months, added to standard-of-care pharmacotherapy; matching characteristics and prohibited/allowed concomitant medications described.
Methods – outcomes	14	Define prespecified primary and secondary outcomes, including variable, analysis metric, aggregation method, and assessment time point.	Methods – Outcomes; Statistical analysis; Study visits and assessments: primary short-term HAMD-17 change at month 2, long-term relapse/clinical worsening over months 4–12, secondary response/remission/continuous outcomes, and safety outcomes are defined with time points.
Methods – harms	15	Describe how harms and unintended effects were defined and assessed.	Methods – Outcomes; Safety analyses; Study visits and assessments; Ethics, funding, and oversight: AEs/SAEs and protocol-defined clinical/laboratory abnormalities, visit/telephone ascertainment, and reporting procedures are described.

Section / topic	Item	CONSORT 2025 reporting requirement	Reported in ASPIRIN BD manuscript / note
Methods – sample size	16a	Explain how sample size was determined and give all assumptions used.	Methods – Sample size: 3-point HAMD-17 MCID, two-sided alpha=0.05, 80% power, 88 required, inflated to 100 for expected attrition.
Methods – interim analyses	16b	Describe interim analyses and stopping guidance, if applicable.	Methods – Changes from protocol-specified statistical analyses: two non-prespecified interim safety reviews were performed under blinding; no formal interim efficacy testing and no change to recruitment, sample size, stopping, procedures, or final analyses.
Methods – randomisation sequence	17a	State who generated the allocation sequence and how it was generated.	Methods – Randomization and blinding: computer-generated block-randomisation sequence is described. The current draft does not explicitly identify the person/role who generated the sequence.
Methods – randomisation restrictions	17b	Describe the type of randomisation and any restrictions such as stratification, blocking, and block size.	Methods – Randomization and blinding: 1:1 block randomisation; block sizes 2, 4, or 6; stratified by centre and baseline HAMD-17 severity (<18 vs >=18).
Methods – allocation concealment	18	Describe how the allocation sequence was implemented and concealed until assignment.	Methods – Randomization and blinding: central IWRS assigned unique identifiers and corresponding pre-labelled study-drug packs; code-based dispensing and identical study packs maintained concealment.
Methods – implementation	19	Clarify whether enrolling staff and staff assigning treatment had access to the allocation sequence.	Methods – Randomization and blinding: allocation was implemented centrally through IWRS; investigators were blinded and dispensing was code-based. Direct access to the underlying allocation list by enrolling/assigning personnel is not explicitly stated.
Methods – blinding	20a	Report who was blinded after treatment assignment.	Methods – Randomization and blinding: participants and investigators were blinded; the statistician analysing study results remained blinded.
Methods – blinding	20b	Explain how blinding was achieved and how similar the interventions were.	Methods – Interventions; Randomization and blinding: placebo and ASA tablets were matched in appearance/organoleptic features; coded central dispensing and emergency-unblinding procedures described.
Methods – statistical methods	21a	Describe statistical methods for primary, secondary, and harm outcomes.	Methods – Statistical analysis: ANCOVA, Pearson chi-square and Fisher exact tests, NRI/LOCF sensitivity analyses, complete-case analyses, exploratory interaction models, and final safety analyses are described.
Methods – analysis populations	21b	Define who was included in each analysis and which assigned group they were analysed in.	Methods – Populations: ITT, mITT, and safety populations explicitly defined; analyses conducted by randomised treatment group.
Methods – missing data	21c	Explain how missing data were handled.	Methods – Handling of missing data: observed-data mITT primary analysis; LOCF ITT sensitivity at V2; NRI for long-term co-primary outcome; complete-case sensitivity analysis; no imputation for secondary or safety outcomes.
Methods – additional analyses	21d	Describe additional analyses (e.g., subgroup/sensitivity) and distinguish prespecified from post hoc/exploratory.	Methods – Sensitivity analyses and Exploratory tertiary analysis: somatic comorbidity; Results – corresponding sensitivity and exploratory analyses.
Results – participant flow	22a	For each group, report numbers randomised, receiving intended intervention, and analysed for the primary outcome.	Results – Participant flow and Figure 1: 121 screened; 100 randomised (50 ASA/50 placebo); all 100 received study medication; V2 primary observed-data analysis included 47 ASA and 48 placebo.
Results – participant flow	22b	For each group, report post-randomisation losses/exclusions and reasons.	Figure 1 and Results – Participant flow: per-visit assessed numbers, cumulative discontinuations, and withdrawal reasons are shown for both arms through V6.
Results – recruitment/follow-up	23a	Report dates of recruitment and follow-up for benefit and harm outcomes.	Methods – Participants; Results – Participant flow: recruitment 15 May 2023 to 31 Dec 2025; study follow-up continued until 31 May 2026; 13-month participant follow-up schedule described.
Results – trial end	23b	If relevant, explain why the trial ended or was stopped.	Trial reached the planned sample of 100 participants and follow-up was completed; manuscript reports no interim decision that changed recruitment, sample size, or stopping. No early termination is reported.
Results – intervention delivery	24a	Report how intervention and comparator were actually delivered, including adherence/fidelity where relevant.	Results – Adherence; Figure 1; Methods – Interventions: pill-count adherence >=80% was 45/47 (95.7%) ASA and 46/48 (95.8%) placebo; MARS remained high and comparable.
Results – concomitant care	24b	Report concomitant care actually received in each group during the trial.	Baseline psychotropic treatment is reported in Table 1 and the protocol for recording treatment changes is described in Methods – Interventions. A consolidated between-group summary of concomitant treatment changes during follow-up is not presented.
Results – baseline data	25	Provide a table of baseline demographic and clinical characteristics by group.	Table 1: baseline demographic, bipolar-disorder, treatment, somatic-comorbidity, and laboratory characteristics by randomised arm.
Results – outcomes/estimation	26	For each primary and secondary outcome, report group-specific numbers analysed/available, results, effect estimates with precision, and both absolute and relative effects for binary outcomes.	Results and Table 2: group-specific denominators and results are provided at each visit; primary continuous and long-term composite outcomes include effect estimates and 95% CIs / ARR / NNT. Secondary categorical outcomes are reported as n/N (%) with between-group tests; relative effect estimates are not reported for every secondary binary outcome.
Results – harms	27	Report harms and unintended effects for each group.	Results – Safety and Table 3: AEs, SAEs, vital signs, weight, ECG, coagulation, bleeding time, laboratory abnormalities, and post-treatment safety are reported by arm.

Section / topic	Item	CONSORT 2025 reporting requirement	Reported in ASPIRIN BD manuscript / note
Results – ancillary analyses	28	Report results of additional analyses, including subgroup and sensitivity analyses, and label them as prespecified or exploratory/post hoc.	Results – Sensitivity analysis; long-term complete-case sensitivity analysis; Exploratory tertiary analysis: somatic comorbidity.
Discussion – interpretation	29	Interpret findings in a way consistent with the results, balancing benefits and harms and considering other evidence.	Discussion – Principal findings; Findings in context of previous evidence; Safety and adherence; Clinical implications and future research; Conclusions.
Discussion – limitations	30	Discuss trial limitations, including possible bias, imprecision, generalisability, and multiplicity where relevant.	Discussion – Strengths and limitations: single dose, restricted safety population, age 18–60, attrition, Poland-only setting, multiple secondary/exploratory comparisons, coded GAF, composite endpoint, blinding integrity, and pending biomarker analyses.

Reference: Hopewell S, Chan A-W, Collins GS, Hróbjartsson A, Moher D, Schulz KF, et al. CONSORT 2025 statement: updated guideline for reporting randomised trials. BMJ. 2025;389:e081123. doi:10.1136/bmj-2024-081123.

Note: CONSORT 2025 supersedes CONSORT 2010. The present checklist is adapted to the current manuscript and should be updated with final page/line numbers if required by the journal at submission.
